# Diabetes Mellitus Family Assessment Instruments: A Systematic Review of Measurement Properties

**DOI:** 10.3390/ijerph20021325

**Published:** 2023-01-11

**Authors:** Vânia Lídia Soares, Sara Lemos, Maria do Céu Barbieri-Figueiredo, Maria Carminda Soares Morais, Carlos Sequeira

**Affiliations:** 1 Institute of Biomedical Sciences Abel Salazar, University of Porto, 4050-313 Porto, Portugal; 2 Center for Health Technology and Services Research (CINTESIS@RISE), 4200-450 Porto, Portugal; 3 Nursing Department, University of Huelva, 21071 Huelva, Spain; 4 School of Nursing of Porto, 4200-072 Porto, Portugal; 5 Centre for Health Studies and Research, University of Coimbra, 3004-504 Coimbra, Portugal; 6 School of Health, Polytechnic of Viana do Castelo, 4900-367 Viana do Castelo, Portugal; 7 Health Sciences Research Unit: Nursing (UICISA: E), 3000-232 Coimbra, Portugal

**Keywords:** diabetes mellitus, family, health literacy, nursing, psychometrics, systematic review

## Abstract

Although many instruments are used to assess the families of people with diabetes, their measurement properties have not been systematically reviewed. We aimed to identify and evaluate the psychometric properties of the instruments used to assess family functioning in adults with diabetes. Methods: A systematic literature review, according to the JBI systematic reviews of measurement properties, was conducted using different databases, including gray literature. PROSPERO registration number: CRD42021239733. Two independent reviewers searched, screened, and assessed the risk of bias among the articles according to the COSMIN methodology. The quality of each included instrument was assessed using the updated criteria for good measurement properties. Results: Eighty-one studies were included, and thirty-one eligible instruments were identified. The psychometric properties frequently assessed were structural validity, internal consistency, and construct validity. Conclusions: Although 31 instruments were included, none of their psychometric properties were scored as “very good”. From the instruments scored as adequate on development and content validity, five stood out for their quality appraisal.. The development of new instruments is not recommended. More studies should be conducted on the existing instruments to assess the less commonly evaluated psychometric properties. Using valid instruments to develop and evaluate interventions is essential to promote health literacy and the effectiveness of diabetes management.

## 1. Introduction

Diabetes Mellitus, from now on referred to as diabetes, is considered to be a global health emergency, affecting approximately 537 million adults [1]. Due to its strong impact at different levels, including personal, social, and health systems, diabetes is presently in the global political agendas, and it is imperative to slow down its epidemic curves and risk factors.

The diagnosis of a chronic disease, such as diabetes, is a critical event with a psychological impact that affects the patient’s personality development and psychosocial functioning, as well as the health of their family members [2].

Being diagnosed with diabetes requires changes in their lifestyle and the integration of complex, long-term, daily self-management routines. In addition, the family, as the primary source of health information, is affected, and this determines how people with diabetes accept the challenges and demands that diabetes imposes [3,4].

Despite their health literacy level and diabetes-specific knowledge, the patient and their family are responsible for disease management [5]. Therefore, family is a crucial source of social support in diabetes day-to-day care.

The importance of social support, namely, familial involvement in diabetes management, has been highlighted in the literature as a determining factor for the patient’s well-being and the success of the different approaches that have been developed [6,7].

Social support can be defined as the existence or availability of people who can help and care when they are needed [8], and it is strengthened by the social bonds between people. This support can be provided by direct family members, peers, friends, neighbors, co-workers, or contacts that are established through social networks [9], and it has led to improvements in the management of diabetes [10,11].

Diabetes requires multidimensional care, and the family is considered to be a care unit [12].

However, it is important to be aware of the numerous changes that have occurred in the concept of family in recent decades. Family is not restricted to blood ties or a legal definition, in contrast, “family is who they say they are” [13] (p. 55).

From the moment of diagnosis, it is essential to care not only for the patient, but also for the family, identifying and understanding the psychosocial factors in diabetes management, including family functioning.

Family functioning is defined as a multidimensional construct, and it comprises two dimensions [14]: family competence (the family structure and capacity to adapt to changes) and family style (the style and quality of family interaction). According to Andrade et al. [15], family functioning can be defined as how members cope with difficulties, conflict situations, means of survival, and role distribution.

In people with diabetes, family functioning is one of the main factors that affects the patient’s quality of life [16], and it is directly associated with the perception that people with diabetes have of their family members’ involvement and support in disease management [17].

Person-centered approaches for people with diabetes allow us to identify the individual and clinical characteristics relevant to the disease, the importance of which is undeniable. However, studies on diabetes and its complications [1,6] have exposed the fragility of these individualized approaches, and there is an emerging need to identify new strategies that are meant to be more effective.

It is essential to empower and promote health literacy, not only people with diabetes, but their families, involving them actively and intentionally in the behavioral changes that the disease requires, which are aiming at the prevention and reduction of complications, as well as assuring an excellent quality of life [11,18].

Studies developed on the family involvement in diabetes are mainly focused on children and/or adolescents with type 1 diabetes and their caregivers, usually the parents. Studies on the families of adult people with diabetes are scarce in the literature [19].

Gaps in the literature are evident in terms of the evaluation and involvement of the family in the process of managing diabetes when the patient is an adult.

Several instruments have been developed and used to assess family functioning, however, they are not specific to families of people with diabetes [19]. Nurses should be encouraged to learn more about family assessment instruments and to make the decision to use one instrument in preference to another in their nursing practice according to the construct of interest and the psychometric properties of each instrument. This will allow researchers to develop interventions adapted to the specifics of each patient and their family, as well as to their context, with a consequential improvement in the health outcomes, namely, health literacy.

A preliminary analysis of the literature allowed us to conduct a systematic review of diabetes-specific family assessment instruments research dating to between 1982 and 2010, which included participants with diabetes of different ages (including children and adolescents) and their families. In this study, the authors consider the importance of professionals deepening their knowledge about the different instruments of family assessment (diabetes specific and generic) and evaluating their effectiveness according to the research questions and objectives of clinical practice [19].

In the preliminary search in the JBI Database of systematic reviews and implementations reports, the Cochrane database of systematic reviews, CINAHL (via EBSCO), the COnsensus-based Standards for the selection of health Measurement INstruments (COSMIN) Database of Systematic Reviews, MEDLINE (via PubMED), Scielo and Prospero, we did not find other types of literature reviews (published or to be developed) in the study area.

Although family assessment instruments have been developed and used on the families of people with diabetes, the assessment of the methodological quality of the studies and the measurement properties have not been reviewed.

This review was conducted to identify and critically evaluate the psychometric properties of the different instruments used to assess family functioning of the families of people with diabetes.

## 2. Methods

The systematic review was developed according to the JBI systematic reviews of measurement properties [20] to answer the following review question: “What are the instruments used to assess family functioning in families with people with diabetes, and what are their psychometric properties?”.

The study protocol was registered in PROSPERO (registration number: CRD42021239733). The review team conducted a thorough analysis of the preliminary searches (prior to the protocol development), and they made a few changes to the protocol before conducting the study selection, however, it is important to point out that the initial focus was maintained.

### 2.1. Search Strategy

A systematic review was conducted using databases and scientific repositories, continuing the study developed by Song et al. [19], and it was limited to studies from 1 January 2010 to 8 June 2021.

According to the JBI recommendations, the search strategy included an initial search for studies published in the PubMed, CINAHL, and Scielo databases using keywords such as “diabetes” OR “diabetic” AND “family” OR “families”, the aim of which was to identify the indexed and free text terms most frequently used in this area of study within titles and abstracts of the studies.

A second search was conducted with the indexed search terms and keywords, which was adapted and individualized to the following electronic databases: MEDLINE^®^, CINAHL^®^, Scopus^®^, Cochrane Library, SciELO—Scientific Electronic Library Online, PsycINFO (access via EBSCOhost Web) and JBI Evidence Synthesis.

Regarding the gray literature, it was mapped using the databases: RCAAP (Scientific Open Access Repository of Portugal) and OpenGrey. The search strategy used for MEDLINE^®^ is provided in Supplementary Material File S1.

In addition, a manual search was performed with the Google Scholar web search using the keywords (diabetes AND “family assessment instruments” OR “social support”). The studies identified were screened manually to check all of the references and to identify the potentially relevant studies. When the studies’ methodology did not properly describe the instruments, the respective validation studies were identified as a source of additional psychometric information, regardless of the year of publication [21,22,23,24,25,26,27,28,29,30,31,32].

The search was performed by two independent reviewers, and it was supported by a librarian.

### 2.2. Study Eligibility Criteria

The studies were eligible if they included any instrument that was developed, tested, or used to assess one or more attributes of family functioning of the families of adult people with diabetes, including published and unpublished primary studies, after 2010, despite the country or setting.

As a source of additional information on the psychometric properties, the development and validation studies of the identified instruments were included regardless of the year of publication.

Studies published in English, Portuguese, Spanish and French were included based on the level of linguistic proficiency of the reviewers, ensuring greater rigor in the selection of evidence and data extraction.

We excluded studies whose methodology was not adequately described and those that did not provide additional information about the instruments’ psychometric properties. Studies were also excluded if the population was under the age of 18 years. Systematic reviews and qualitative studies were also excluded.

### 2.3. Study Selection

This process was performed by two reviewers, and it is presented and organized in a PRISMA flow diagram [33].

The articles were screened for inclusion using the Endnote X9^®^ Software reference management software (Clarivate Analytics, Philadelphia, PA, USA), and duplicate references were identified and removed. Two authors independently screened the titles and abstracts. The same two authors assessed the potentially relevant full-text information. Disagreements between the two reviewers at any stage of this process were resolved by coming to a consensus or through performing an analysis and having a discussion with a third reviewer.

### 2.4. Assessment of Methodological Quality and Assessment of Measurement Properties

The methodological quality of the studies was assessed through the COSMIN guideline for systematic reviews, which was developed specifically for patient-reported outcomes measures (PROMs), which can also be used in other types of measurement instruments [34].

The systematic review only included studies satisfying the criteria of the methodological quality assigned to each study using the COSMIN Risk of Bias checklist [34].

The ten boxes of the COSMIN include: (1) instrument development; (2) content validity; (3) structural validity; (4) internal consistency; (5) cross-cultural validity; (6) reliability; (7) measurement error; (8) criterion validity; (9) hypothesis testing; (10) responsiveness. Each standard was rated on a four-point rating system as ‘very good’, ‘adequate’, ‘doubtful’, or ‘inadequate’. The overall quality rating for each study was determined by taking the lowest rating on an item of any box [34,35,36].

The content validity, which is considered to be the most important measurement property, was evaluated [36].

To assess the quality of each instrument regarding the measurement property, the updated criteria for good measurement properties was applied [34].

The results were rated as sufficient (+), insufficient (−), or indeterminate (?), and the principle of “the worst score counts” was applied.

Using the COSMIN methodology, we defined the hypotheses in advance to evaluate the results of studies on construct validity:

- Correlations with instruments used to measure the same construct should be >0.50;
- Correlations with instruments used to measure related constructs should be in the range of 0.30–0.50.

Two reviewers performed each step-in assessment of the methodological quality, and the results were obtained by coming to a consensus.

### 2.5. Evidence Synthesis

The evidence synthesis was conducted according to the COSMIN methodology [34,35,36]. The key characteristics of each instrument tested, the COSMIN Risk of Bias checklist results for the instrument development, the content validity, the others measurement properties, and the results of each study on each measurement property have been rated and summarized in tables.

Relevant information from all of the included studies is described in Supplementary Material File S2. The summary of the principal results on the measurement properties of each instrument described in the different studies is reported in Supplementary Material File S3.

## 3. Results

### 3.1. Studies Selection

**Figure 1:**
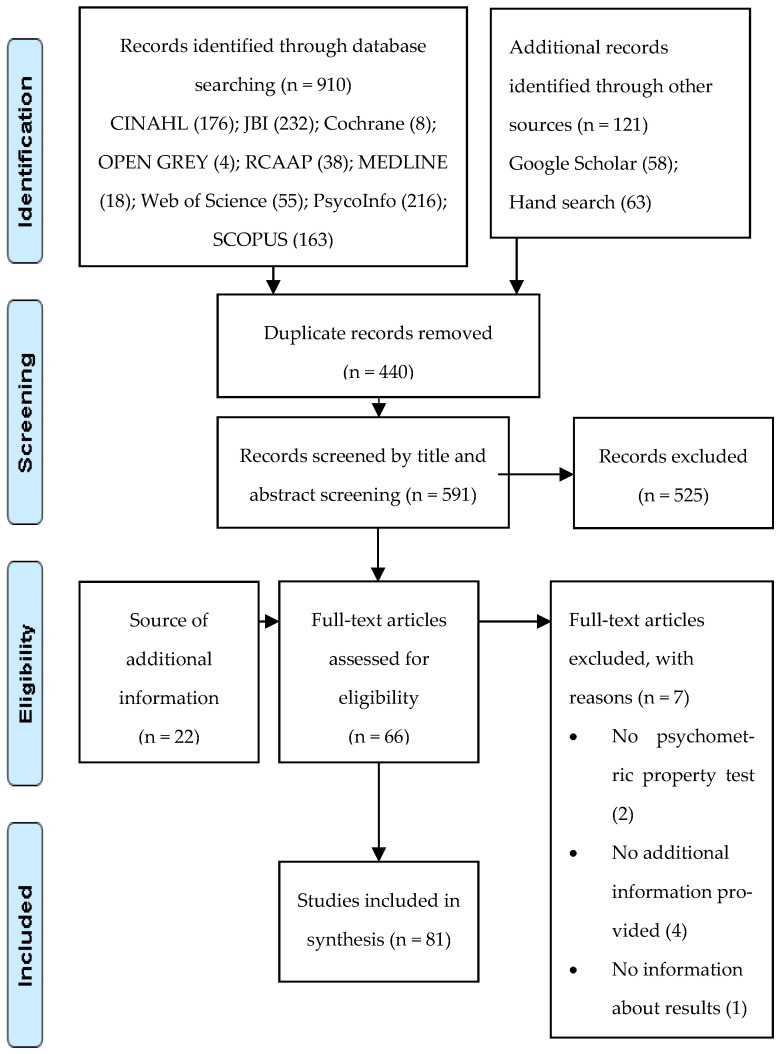
PRISMA flow chart of study selection.

The search strategy identified 1031 studies. After removing the duplicates, 591 articles were screened by the relevance of their titles and abstracts. The remaining 66 full-text articles were assessed for eligibility, and seven of them were excluded for different reasons. As an additional source of psychometric information, 22 additional articles were identified through reference screening. A total of 81 articles were included in this systematic review, and 31 family assessment instruments were identified (Figure 1).

### 3.2. Study Characteristics

In this systematic review, 39 studies were conducted in the USA. The remaining 42 studies were conducted in 24 different countries.

The study design that was mainly used was cross-sectional. The sample size ranged from 48 to 8596 participants. The community setting was reported in 62 studies. Other settings included hospitals and diabetes centers.

The majority of the studies did not describe the concept of family. Eight studies focused on the concept of family in terms of the patients’ marital status, blood ties, close relatives, or living with patients, and another eight studies had a broad approach to the concept of family.

In the Supplementary Material, File S2 presents the relevant information of each included study, and File S3 summarizes the results of the measurement properties analysis of each instrument described in the different studies that were included.

### 3.3. Instruments Characteristics

The review identified 31 instruments; 15 of them were specific instruments used to assess the families of people with diabetes. Table 1 summarizes the information for each instrument included (diabetes-specific and generic ones).

The type of measure was mostly self-reported. Each instrument reported different constructs of family functioning. As expected, the specific instruments assessed the attributes/actions and family dynamics, and they focused more on diabetes management and the disease challenges. The generic instruments focused more on the families’ strengths, weaknesses, values, and behaviors during daily life problems and when facing a problem/adversity.

The Diabetes Family Behavior Checklist (diabetes-specific instrument) and the Family APGAR Index (generic instrument) were the most reported instruments. 

### 3.4. Methodological Quality of Included Studies

The psychometric properties most frequently assessed in each study were structural validity, internal consistency, and construct validity (Supplementary Material File S3).

Of the eighty-one articles included, twenty-seven articles were validation studies, including nineteen original validation studies and eight cultural and linguistic adaptation studies.

The remaining studies did not report sufficient data on the psychometric properties, therefore, it was necessary to include 22 studies as a source of additional information.

Table 2 shows the results of quality assessment of each instrument on the development and content validity using the COSMIN Risk of Bias checklist. The other measurement properties results are displayed in Table 3.

Ten studies presented information about the instrument development, which was scored as adequate, and twelve studies had a doubtful score. The remaining studies did not report information about the instrument development.

According to the COSMIN Risk of Bias checklist, six studies were assigned an adequate score based on the content validity, and fourteen studies had a doubtful score. The remaining studies did not report information on content validity.

The structural validity was assessed in twenty-seven studies through a confirmatory or exploratory factor analysis: twelve studies were rated as very good in terms of structural validity, fourteen were rates as adequate, and one was rates as inadequate.

The internal consistency was assessed in 63 studies and, for the most part [56], the authors reported this measurement property using Cronbach’s alpha values. One study used the reliability coefficient. Internal consistency was generally scored as very good, except in one study that was scored as insufficient, in which the authors only reported Cronbach’s alpha value of the total scale.

Reliability was assessed using the test–retest methodology in seventeen studies and through correlation coefficients in three studies. The other studies did not include the assessment of reliability. By considering the time of two weeks as an appropriate time interval, according to similar studies [100], twelve studies were rated as very good, and four studies were rated as doubtful. One study did not state the time interval. Three studies were rated as inadequate because the interval was not appropriate.

Family functioning is a multidimensional construct, therefore, the literature does not recognize a gold standard instrument that can be used to assess this construct. However, ten studies assessed the concurrent validity comparing the instrument to other well-established instruments that assess similar constructs. In these studies, the criterion validity was rated as very good.

Hypotheses testing for construct validity was assessed by comparing it with other similar outcome measurement instruments in 12 studies and by comparing between the subgroups in eight studies. Only one study was rated as doubtful due to it having a poor description of the characteristics of the subgroups. The remaining studies were scored as adequate, as not all of the important characteristics of the subgroups had been described, and there were doubts about whether the comparator instrument was appropriate for the study population or not.

Responsiveness was reported in one study using a construct approach (before and after the intervention), and because there was a poor description of the intervention, it was rated as doubtful.

No other psychometric characteristics were described.

### 3.5. Quality of Psychometric Properties of Instruments

The measurement properties for each instrument were described and evaluated according to the updated criteria for good measurement properties [34], and they are presented in alphabetic order in Table 4.

Brief Family Assessment Measure (Brief FAM-III): This instrument was used in two studies [64,65]. One study [65] reported the structural validity, which was rated as insufficient due to the absence of criteria for a sufficient status, and the internal consistency rated as sufficient.

Chronic Illness Resources Survey (CIRS): Internal consistency was rated as insufficient in both of the studies that reported this instrument [66,67]. These results were explained by Cronbach’s alpha value ≤ 0.70 [67] and the missing reported values of this measurement property in the instrument’ subscales [66]. One of the studies [67] reported the reliability rated as sufficient for correlations with other instruments for related constructs > 0.30. The construct validity was rated as indeterminate due to it having insufficient information.

Diabetes Caregiver Activity and Support Scale (D-CASS): This instrument was reported in one study [37]. Structural validity was assessed through exploratory factor analysis, and it missing other information meant that was rated as insufficient. Internal consistency and reliability were rated as sufficient.

Diabetes Care Profile (DCP): Six studies reported this instrument [7,38,39,40,41,42]. Structural validity was described in one study [39], and it was rated as indeterminate for having insufficient information. Also, the criterion validity was scored as insufficient for the correlations values. The construct validity was scored as sufficient. Three studies reported internal consistency, two studies were rated as sufficient [38,41], and one study was as insufficient [39] for Cronbach’s alpha value ≤ 0.70 in the subscales.

Diabetes Family Behavior Checklist (DFBC): This instrument was tested in seven studies [31,43,44,45,46,47,48]. One study’s reported structural validity was rated as indeterminate due to it lacking information [45]. The internal consistency was reported in six studies. Four studies were scored as sufficient [44,45,47,48], and two studies were scored as insufficient [31,43] as they had Cronbach’s alpha values ≤ 0.70. The hypothesis testing for construct validity was reported in one study [31], and it was rated as insufficient, which was justified by the correlations values (r < 0.30).

Diabetes Family Behavior Checklist-II (DFBC-II): Five studies tested this instrument [29,49,50,51,52]. One study [29] assessed the internal consistency and reliability. The internal consistency was rated as insufficient (Cronbach’s value in one subscale < 0.70). Reliability was scored as indeterminate when the studies were missing Intraclass Correlation Coefficient (ICC) values. The internal consistency, which was reported in two studies [49,50], was rated as sufficient.

Diabetes Family Support and Conflict Scale (DFCS): Two studies reported the structural factor validity [53,54]. One study was rated as sufficient [54], and the other one was rated as insufficient [53] for lacking all of the relevant information. The internal validity was reported as sufficient in both of the studies. The reliability was scored as indeterminate in Sofulu et al. study because it contained insufficient information [54].

Diabetes Mellitus 2 Treatment Adherence Scale version III (EATDM-III): this scale was reported in one study [55]. The exploratory factor analysis was performed and scored as insufficient due to it having missing information. The internal consistency was scored as sufficient.

Diabetes Support Scale (DSS): This scale was tested in two studies [28,29,30,31,32,33,34,35,36,37,38,39,40,41,42]. One study [28] described the internal consistency score as sufficient. For the low criterion validity, the correlation value (r < 0.70) was rated as insufficient. Similarly, the construct validity was scored as insufficient (r < 0.30).

Empowerment Questionnaire: This scale was evaluated in one study [46]. The internal consistency was rated as sufficient. The structural validity and construct validity were scored as indeterminate due to it having insufficient information. The reliability was scored as insufficient (r values ≥ 0.56).

Family Adaptability and Cohesion Evaluation Scale (FACES IV): This instrument was tested in three studies [68,69,70]. Only one of these studies reported the structural validity, internal consistency, criterion validity, and construct validity; all of them were scored as sufficient [69]. Another study reported the internal consistency, and we scored it as sufficient [68].

Family APGAR Index: The measurement properties of this instrument were reported in two out of the seven studies [15,30,32,71,72,73,74] that used this instrument. One study [30] assessed the internal consistency, and we rated it as insufficient (Cronbach value < 0.70), and the construct validity score was sufficient. Another study [32] reported internal consistency and reliability, and we scored both as sufficient.

Family Assessment Device (FAD): This scale was reported in five studies [16,27,75,76,77]. The internal consistency was rated as sufficient in three studies [16,27,75], whereas it was as insufficient in the study of He et al. [76] due to it having alpha de Cronbach’ values of 0.53. The reliability was scored as indeterminate because it did not describe the ICC values [76]. In the study by Epstein et al. [27], for correlations values < 0.70, the criterion validity was considered to be insufficient.

Family-Carer Diabetes Management Self-Efficacy Scale (F-DMSES): One study evaluated this scale [56] and described the measurement properties. The internal consistency was scored as sufficient. The structural validity (lack of criteria for sufficient), the reliability (ICC value < 0.70), and the criterion validity (r < 0.70) were scored as insufficient.

Family Emotional Involvement and Criticism Scale (FEICS): This scale was tested in three studies [24,58,78]. Two studies reported the internal consistency, and we scored it as sufficient [24,78]. One study [24] assessed the structural validity, which we rated as insufficient. The same study was scored as sufficient in terms of construct validity and as insufficient in terms of criterion validity (r < 0.70).

Family and Friend Involvement in Adults’ Diabetes (FIAD): Two studies used this instrument [57,58]. One study reported the internal consistency’ value, which we scored as sufficient [58]. The other study [57] reported the structural and construct validity, and both of the properties were rated as sufficient. This study also assessed the internal consistency, criterion validity, and reliability, which were all rated as insufficient (values of each measurement property reported < 0.70).

Family Function Questionnaire (FFQ): This instrument was tested in two studies [17,79]. One study [79] reported the internal consistency and reliability, which we rated as insufficient due to it having low values (Cronbach’s alpha and ICC < 0.70). However, the construct validity was scored as sufficient. The study by Pamungkas and Chamroonsawasdi [17] assessed the internal consistency, which we rated as sufficient.

Family Functioning Style Scale: Two studies described this instrument [80,81]. Only one study [81] assessed the measurement properties. The internal consistency was rated as sufficient, and the structural validity was rated as indeterminate, which were justified by the paper lacking information.

Family Support Scale adapted for African American women with type 2 Diabetes Mellitus (FSS-AA): The study by Littlewood et al. [59] reported internal consistency, which was scored as sufficient. The other measurement properties, the structural validity (criteria for sufficient not reported), reliability (ICC value < 0.70), criterion validity (correlations < 0.70), and construct validity (r < 0.30), were rated as insufficient.

Helping for Health Inventory Couples version (HHI-C): One study [60] described this instrument. The internal consistency and criterion validity were described, and we scored them as insufficient (Cronbach value in one of the subscales and correlations values were both <0.70). The reliability was scored as indeterminate (missing values). The construct validity was rated as insufficient (r < 0.30).

Instrumental Expressive Social Support Scale (IESS): This scale was reported in two studies [82,83]. One study [83] described the internal consistency, and we scored it as sufficient. The other study [82] reported the structural validity (rated as insufficient as criteria for sufficient not met), and the internal consistency was scored as sufficient.

Important Other Climate Questionnaire (IOCQ): Two studies tested this instrument [58,84]. The internal consistency was reported, and we scored as sufficient in both of the studies. Only one study [84] described the structural validity (rated as sufficient), and the construct validity was rated as insufficient due to it having low correlations values (r < 0.30), and the reliability was rated as insufficient due to it having missing information.

Multidimensional Diabetes Questionnaire (MDQ): The internal consistency was reported, and we scored it as sufficient in the two studies that tested this instrument [25,61]. The structural validity was rated as sufficient in study by Talbot et al. [25] and, in contrast, the reliability was rated as indeterminate due to it missing information.

Multidimensional Scale of Perceived Social Support (MSPSS): Four out of six studies [26,72,85,86,87,88] that used this instrument, and we scored the internal consistency as sufficient [26,85,86,87]. Two studies [26,86] assessed the structural validity scored as insufficient (criteria for sufficient not met). The reliability was evaluated by Zimet et al. [26], and we scored it as indeterminate (values not reported). One study also assessed the criterion validity and the construct validity [86]: the first one was rated as insufficient (values of correlations < 0.70, and the second one was scored as sufficient.

Patient Assessment of Chronic Illness Care-Short Form (PACIC-SF): Only one study of the four studies [89,90,91,92] described the psychometric properties. This study [89] reported the internal consistency and construct validity, and both of them were rated as sufficient, and the structural validity was scored as insufficient (criteria for sufficient not described).

Perceived Social Support from Friends (PSS-Fr) and from Family (PSS-Fa) scales: The internal consistency was reported, and we rated it as sufficient in two studies [22,93]. However, the structural validity was reported in only one study [22], and we rated as indeterminate due to it lacking information.

Perceptions of Collaboration Questionnaire (PCQ): This instrument was tested in two studies [58,94]. One study [94] was assessed and the structural validity (criteria for sufficient not described) and the internal consistency (Cronbach’s alpha value < 0.70) were scored as insufficient. The study by Mayberry et al. [58] described the internal consistency, which as rated as insufficient (Cronbach’s alpha value in one subscale < 0.70).

Scales to measure social support for diet and exercise behaviors: The internal consistency was described in the five studies that tested this scale [23,93,95,96,97]. Three studies were rated as sufficient [93,96,97] and the remaining two studies were rated as insufficient as the reported Cronbach’s values were < 0.70 [23,95]. The construct validity was reported in one study [23] and it was scored as insufficient (correlations’ values < 0.30). These authors also reported the structural validity (rated as insufficient for having insufficient criteria), criterion validity (rated as insufficient for having a correlation´s value < 0.70), and reliability (rated as indeterminate for missing values of ICC).

Social Provisions Scale (SPS): The internal consistency was reported, and we rated it as insufficient in the three studies that tested this instrument for having subscale Cronbach’s values < 0.70 [21] and for having missing information [98,99]. Cutrona and Russell [21] also described the structural validity, and we rated it as insufficient due to the absence of information, and we rated the construct validity as sufficient.

Social Support Scale for Self-care in Middle-Aged Patients (S4-MAD): Naderimagham et al. [62] developed this instrument and reported structural validity, internal consistency, and reliability. All of the measurement properties were rated as sufficient.

Unsupportive Social Interaction Scale (USIS): The study by Baron-Epel et al. [63] reported the internal consistency, which we scored as sufficient. Contrarily, the structural validity was rated as insufficient due to it lacking information, and the reliability was indeterminate due to it lacking an ICC value.

## 4. Discussion

This study was conducted to systematically assess the psychometric quality of the instruments developed and used to assess the family functioning of the families of people with diabetes.

We identified 31 instruments focused on different constructs of family functioning which were used in different settings. The majority of the instruments were used in multiple studies, with the exception of eight instruments that were tested in only one study.

This review identified 49 studies that reported insufficient data on the instrument measurement properties. This lack of information led to the need to screen 22 additional sources of psychometric information using the references provided by the authors of the studies. Nevertheless, the studies developed by Takenaka et al. [70] and Regufe [83] were exceptions. In these two studies, the full-text instrument validation was not available to be consulted. For that reason, the original version of FACES IV [69] and the validation study of the IESS, which was also developed in Portugal [82] were retrieved.

None of the psychometric properties of the included instruments in this review were scored as “very good”. This can be explained by the them missing reported values or lacking criteria described in the COSMIN Risk of Bias checklist [34].

Terwee et al. [36] considered content validity to be the most important measurement property when one is selecting an instrument. However, we observed that the content validity was inadequately reported or not reported, except in the validation studies. Only five instruments were rated as adequate in terms of the content validity (Brief FAM-III, DFSC, FIAD, Scales to measure social support for diet and exercise behaviors, and S4-MAD). We found instruments that were developed from other(s) instrument(s), and for that reason, some information was not clearly or sufficiently reported (DFBC, DCP, and Empowerment questionnaire).

Furthermore, the sources of additional psychometric information identified also missed information about content validity and instrument development according to COSMIN criteria.

Most of the instruments were scored as doubtful for content validity due to them lacking information or because it was not clear if the professionals were asked about the relevance and comprehensiveness of the items and/or if the patients were asked about comprehensiveness and comprehensibility.

In addition, some essential psychometric properties were not tested for many of the instruments, or they were rated as insufficient. For most of the studies, the structural validity was not reported, and when it was reported, only seven studies met all of the criteria for a “sufficient” score.

Some studies were rated as insufficient in terms of the internal consistency. This score can be explained because the authors only reported Cronbach’s alpha values for the total scale, missing the subscales or the values [66] or for the correlation´s value < 0.70, e.g., the studies by Fitzgerald et al. [39]; Glasgow et al. [67]; Mayberry et al. [58]. Cross-cultural validity and measurement errors were not reported anywhere.

Of the twenty studies that reported reliability, only five of them were scored as “sufficient”.

Family functioning is a multidimensional construct, and it is associated with the people with diabetes’ perception of their family’s involvement and support [17], which may explain the diversity of the constructs assessed in each identified instrument. Regarding the specific instruments used to assess the families of people with diabetes, the majority of them focused on specific aspects of diabetes management and the social and psychological factors related to diabetes. These instruments seem to be helpful for researchers who want to investigate the effectiveness of a specific intervention in diabetes-related family behaviors through a longitudinal design. Concerning the generic instruments, the constructs were focused on family adaptability, strengths and weaknesses, different types of social support, perceived social support, and family communication, and coping strategies.

By recognizing the family support as being crucial in diabetes care, some instruments (e.g., DSS; FSS-AA) have been developed and used to assess only the supportive behaviors in the families of people with diabetes. However, people with diabetes described sabotaging their family’s behaviors, most commonly regarding diet and exercise [51]. In comparison, other instruments were developed to assess not only the support, but also the non-supportive behaviors (DFBC), the family conflict (DFCS), the helpful and harmful involvement (FIAD), partner investment and resistance (HHI-C), or only the unsupportive social interactions (USIS).

Thus, nurses should recognize that the family environment and their involvement may not always have a health-promoting impact on diabetes and the respective outcomes [53]. The helpful and harmful aspects of family/friend involvement and supportive and non-supportive behaviors should be considered when the family is assessed [57].

Although most of the instruments included in this review, disease-specific or generic ones, were multidimensional and developed to assess multiple family attributes, some instruments are focused only on assessing one specific family attribute, such as the marital dyad, criticism, emotional involvement, or treatment adherence. These instruments are described as unidimensional (e.g., D-CASS and PACIC-SF).

The DFBC was the disease-specific instrument that was most frequently reported, and it targets not only the patient, but also the family members. This instrument was composed by two subscales (supportive and non-supportive family behaviors). The Family APGAR Index was the generic instrument that was most frequently tested.

Regarding the overview of the quality appraisal of all of the instruments in terms of development and content validity and considering the instruments that were scored as adequate in terms of both of them according to the COSMIN methodology, these allow us to highlight the Brief FAM-III, the DFSC, FIAD, the Scales to measure social support for diet and exercise behaviors and S4-MAD instruments.

The Scales to measure social support for diet and exercise behaviors, for its specificity, although it is very important in families with people with diabetes, could be insufficient in recognizing and understanding the family functions that promote the family involvement in the behavioral challenges that the disease requires.

The S4-MAD scale was developed to assess the self-care social support in diabetic patients that are middle ages, considering that most of the patients are this age. This instrument emphasizes the importance of all forms of social support in diabetes management and metabolic control. However, it is important to mention that social support in general, which is recognized in the literature as a determining factor for the success of the different approaches developed with people with diabetes and their families, can also be a source of conflict and stress [53].

Thus, the other scales can be used to assess important family’ behaviors: diabetes-specific instruments allow us to assess the helpful and harmful aspects of family and friend involvement in the lives of adults with diabetes (FIAD) and the supportive and non-supportive behaviors of families with people with diabetes (DFSC).

Other instruments emphasize the other dimensions of social support, and for that reason, they can be considered to be valid options for assessing family functioning from a more holistic perspective. The Brief FAM-III scale allows us to perform an assessment of the family functioning from different perspectives (the family as a system, the relationships between specific pairs in the family, and the individual’s perception of their functioning in the family). Although it is a generic instrument, it allows us to identify the family’s perceptions of the strengths and weaknesses in their family functioning.

Diabetes is described as “a pandemic of unprecedented magnitude which is spiraling out of control” [1]. The International Diabetes Federation reports a global increase in diabetes prevalence, and this presents a challenge to the health and well-being of people with diabetes, family, and society [1]. This information has exposed the fragility of the actual approaches and the importance of identifying new strategies that are meant to be more effective in controlling and managing diabetes.

The interventions should be developed for people with diabetes and their family and according to the specificities of their social and living situations.

Therefore, in families with people with diabetes, it is crucial that nurses identify, through valid instruments, the relationships established between the family members and their behaviors. As it has already been mentioned, not all of the support and involvement from family is perceived as positive by people with diabetes. Nurses should recognize behaviors that are an incentive, obstacles, or barriers to disease management [4] assessing family and promoting their involvement in diabetes challenges.

However, it is recommended that health professionals do not develop more instruments, but instead, they should invest in deepening their knowledge of the existing instruments, their characteristics, and their psychometric properties.

Therefore, the decision to use one instrument should be made based on a quality appraisal of the measurement properties of the instrument and by taking into consideration the country, the target population, and the goals of the study.

### Strengths and Limitations

We have developed an extensive systematic review of family functioning instruments in families with people with diabetes, including grey literature. Regardless of our decision to limit the search strategy to 2010, by continuing another study in this area [19], we performed reference screening, irrespective of the year of publication, as a source of additional information. This fact is considered to be a strength of this study by the authors.

We considered the COSMIN guidelines and updated the quality criteria for good psychometric properties to assess the quality of each study and each measurement property [34,35,36], which is an important strength of this study.

Most of the studies included in this systematic review were conducted before the establishment of COSMIN guidelines, thus, some measurement properties and instrument development criteria were not reported in these studies. This can justify some of the results presented in the rating scores summary. Another important fact to note is the differences between the countries in their guidelines to assess the measurement properties, and in this review, we included studies from different countries.

By considering family functioning as a multidimensional construct, we may have made errors in the study identification, given the number of results obtained.

Another potential limitation of this study is the variety of studies and instruments that we included. We described 31 instruments to assess the family functioning of families of people with diabetes, which were developed and tested in different countries, settings, and among different target populations.

## 5. Conclusions

This systematic review provides an overview of the instruments that are currently used to assess the functioning of the family of people with diabetes, and it reports their measurement properties and ratings. It can be an important resource to help nurses to select the adequate instrument for their clinical practice and research in different settings and according to the construct that is being assessed.

Although 31 instruments were included, none of their psychometric properties were scored as “very good” according to the COSMIN Risk of Bias checklist. Considering the instruments that were scored as adequate in terms of development and content validity, five instruments were highlighted according to the updated criteria for having good measurement properties.

The development of new instruments is not recommended. Instead, more studies should be conducted on the existing instruments to assess the less frequently evaluated psychometric properties, namely, reliability, measurement error, and responsiveness.

The target population of the studies should consider a diversity of family members, thus adapting to the evolution of the concept of family by not only restricted it people with blood ties.

Using valid instruments to develop and evaluate interventions in families with diabetes is essential to promote health literacy, namely, critical literacy and the effectiveness of diabetes management.

## Figures and Tables

**Table 1:** Summary information of each instrument (Diabetes Mellitus specific and generic) in alphabetical order.

Instrument	References	Type of Measure	Target Population (According to the Validation Study)	Construct(s)	Subscales, Number of Items	Response Options	Theoretical Background
Diabetes Mellitus-Specific Instruments							
Diabetes Caregiver Activity and Support Scale (D-CASS)	[37]	Patient interview	Family. Target family caregivers of persons with T2DM	Caregiver perceptions of difficulty with activities and supportive behaviors specifically while providing care for a person with T2DM	11 items Unidimensional scale	7-point Likert scale	Literature Review
Diabetes Care Profile (DCP)	[7,38,39,40,41,42]	Self-reported	Patients/Target patients with diabetes	Social and psychological factors related to diabetes and its treatment	234 items with 16 scales (control problems, social and personal factors, positive attitude, negative attitude, self-care ability, importance of care, self-care adherence, diet adherence, medical barriers, exercise barriers, monitoring barriers, understanding management practice, long-term care benefits, support needs, support, and support attitude)	Filling in the blanks with the correct answers or by choosing the single best answer	Diabetes Educational Profile and Health Belief Model
Diabetes Family Behavior Checklist (DFBC)	[31,43,44,45,46,47,48]	Self-reported	Patient/family member dyad. Target insulin-dependent Diabetes Mellitus (IDDM) patients and families	Supportive and non-supportive family behaviors specific to the diabetes self-care regimen (drug therapy, blood glucose measurement, exercise, and diet)	16 items that can be divided into 2 subscales for analysis (positive feedback—9 questions; negative feedback—7 questions)	5-point Likert scale	Literature review including information from people diagnosed with diabetes and a health professional who is familiar with the disease
Diabetes Family Behavior Checklist-II (DFBC-II)	[29,49,50,51,52]	Self-reported	Patients/family member dyad. Target patients with type 2 diabetes	Family member’s actions toward the person with type II diabetes (medication taking, glucose testing, exercise, and diet)	DFBC-II version for a partner or significant other: 17 items that can be divided into 2 subscales for analysis (positive feedback—9 questions; negative feedback—7 questions) open-ended item DFBC-II version for diagnosed person: 17 items and a new section designed to assess 17 family behaviors	5-point Likert scale 7-point Likert scale (DFBC-II section for diagnosed person)	Literature Review and Diabetes Family Behavior Checklist (DFBC)
Diabetes Family Support and Conflict scale (DFSC)	[53,54]	Self-reported	Patients. Target patients with type 2 DM	Diabetes-related family support and conflict	10 items including 2 subscales (supportive—6 items; unsupportive—4 items)	4-point Likert scale	Literature review and information from healthcare professionals and patients with diabetes
Diabetes Mellitus 2 treatment adherence scale version III (EATDM-III)	[55]	Self-reported	Patients. Target patients with type 2 diabetes	Treatment adherence	30 items including 6 subscales (physical exercise, family support, medical control and treatment, community support and organization, diet, and information)	5-point Likert scale	Literature Review and subjective reports from providers and patients
Diabetes Support Scale (DSS)	[28,42]	Self-reported	Patients. Target patients with diabetes (type 1 and 2)	Diabetes social support	12 items including 3 subscales (emotional support, advice, information)	7-point Likert scale	Literature Review and rational-theoretical approach
Empowerment questionnaire	[46]	Self-reported	Patients. Target patients with type 2 diabetes	Empowerment of type 2 diabetes patients (based on self-managed dietary/exercise behaviors, psychological impact, and family support)	31 items including 5 scales (self-managed dietary behaviors, self-managed exercise behaviors, psychological impact of diabetes, and positive and negative feedback in patient-family communication) and 13 questions on background	5-point Likert scale Filling in the blanks with the correct answers	Literature review and Japanese-language versions of the Appraisal of Diabetes Scale and Diabetes Family Behavior Checklist
Family-Carer Diabetes Management Self-Efficacy Scale (F-DMSES)	[56]	Self-reported	Families. Target family of patients with type 2 diabetes	Family-caretaker diabetes management and self-efficacy.	14 items including 4 subscales (General diet and blood glucose monitoring, medication and complication, diet in different situations, weight control, and physical activities)	5-point Likert scale	Diabetes Management Self-Efficacy Scale (DMSES)
Family and Friend Involvement in Adults’ Diabetes (FIAD)	[57,58]	Self-reported	Patients. Target patients with type 2 diabetes	Family/friend involvement	16 items including 2 subscales (helpful involvement, harmful involvement)	5-point Likert scale	Literature review, cognitive interviews, and expert input
Family Support Scale adapted for African American women with type 2 Diabetes Mellitus (FSS-AA)	[59]	Self-reported	Patients. Target women with uncontrolled T2DM	Diabetes-specific social support	16 items including 3 subscales (parent and spouse/partner support, community and medical support, extended family and friends support)	5-point Likert scale	Original Dunst Family Support Scale (FSS)
Helping for Health Inventory: Couples Version (HHI-C)	[60]	Patient interview	Patients. Target patients with type 2 diabetes	“Miscarried helping” in couples	15 items including 3 subscales (Conflict/Blame, Partner Investment, Resistance)	5-point Likert scale	Theoretical concept of “miscarried helping” and “Helping for Health Inventory” scale
Multidimensional Diabetes Questionnaire (MDQ)	[25,61]	Self-reported	Patients. Target patients with non-insulin-dependent diabetes	Patients’ psychosocial adjustment to diabetes	41 items grouped into three sections: Section I: 16 items including 3 scales (perceived interference caused by diabetes to daily activities, work, and social and recreational activities; perceived severity of diabetes; perceived diabetes-related social support from a significant other, family, friends, and health professionals) Section II 12 items including 2 scales (positive reinforcing behaviors and specific form of non-supportive behaviors) Section III 13 items including 2 scales (self-efficacy expectancies and outcome expectancies)	Section I and II: 7-point Likert scale Section III: rated from 0 to 100	Literature Review and Social Learning Theory
Social Support Scale for Self-care in Middle-aged Patients (S4-MAD)	[62]	Patient interview	Patients. Target patients with diabetes	Social support for self-care in diabetic patients	30 items including 5 subscales (nutrition, physical activity, self-monitoring of blood glucose, foot care, and smoking)	5-point Likert scale	Literature review
Unsupportive social interaction scale (USIS)	[63]	Patient interview	Patients. Target patients with diabetes	Unsupportive social interactions	15 items including 2 scales (interference and insensitivity)	5-point Likert scale	Literature review and information from patients with diabetes
Generic instruments							
Brief Family Assessment Measure-Brief (Brief FAM-III)	[64,65]	Self-reported	Patients. Target patients with type 2 diabetes	Family’s strengths and weaknesses	42 items including 3 scales (general, dyadic relationships, and self-rating)	4-point Likert scale	Model of Family Functioning
Chronic Illness Resources Survey (CIRS)	[66,67]	Self-reported	Patients. Target post-menopausal women with type 2 diabetes	Multilevel support resources from proximal support (e.g., family and friends) to more distal factors (e.g., neighborhood or community)	22 items including 9 subscales (personal; family and friends; physician/health care team; neighborhood/community; organizations; work; media and policy; dietary and physical activity)	6-point Likert scale	Multilevel, Social–Ecological Model
Family Adaptability and Cohesion Evaluation Scale (FACES IV)	[68,69,70]	Self-reported	Adults. Target Students and nonclinical family	Family functioning (family adaptability and cohesion)	42 items including 2 Balanced scales (cohesion and flexibility) and 4 Unbalanced scales (disengaged, enmeshed chaos, and rigid)	5-point Likert scale	Circumplex model and family therapists fromAmerican Association for Marriage and Family Therapy
Family APGAR Index	[15,30,32,71,72,73,74]	Self-reported	Clinical group(patients)/nonclinical group dyad. Target “normal” families’ members and psychiatric outpatients	Global family function	5-item questionnaire that is designed to test five areas of family function (adaptation; partnership; growth, affection, and resolve) Unidimensional scale	3-point Likert scale	Literature review
Family Assessment Device (FAD)	[16,27,75,76,77]	Self-reported	Students and patient’s family. Target psychology students and a group of patient’s family	Family functioning	53 items (currently is in use a 60-items version) including 7 scales (general functioning, communication, affective involvement, roles, problem solving, affective responsiveness, behavior control)	4-point Likert scale	McMaster Model of Family Functioning (MMFF)
Family Emotional Involvement and Criticism Scale (FEICS)	[24,58,78]	Self-reported	Patients. Target patients receiving primary medical care	Perceived family criticism and emotional involvement	2 subscales (Family’s Perceived Criticism—7 items; Intensity of Emotional Involvement—7 items)	5-point Likert scale	Expressed Emotion (EE) theory
Family Function Questionnaire (FFQ)	[17,79]	Self-reported	Families. Target caregivers of psychiatric patients	Perceived family function	24 items including 3 subscales (problem-solving, communication, and personal goal)	4-point Likert scale	Literature Review and Cognitive-behavioral Family Treatment
Family Functioning Style Scale	[80,81]	Patient interview	Mother/father dyad. Target families	Family functioning style	22 item questionnaire scale (the original scale developed by Deal, Trivette and Dunst, 1988, use a 26-item version) including 3 subscales (interactional patterns and family values, family commitment, intrafamily coping strategies)	5-point Likert scale	Family Functioning Style Model
Instrumental Expressive Social Support Scale (IESS)	[82,83]	Self-reported	Older people. Target community-dwelling older	Social Support	16 items (the original scale used a 20 items version) including 3 subscales (familiar and socio-affective support, sense of control, and financial support)	5-point Likert scale	Literature Review
Important Other Climate Questionnaire (IOCQ)	[58,84]	Self-reported	Patients. Target smokers	Perceived autonomy supportiveness of an “important other”	6 items Unidimensional	7-point Likert scale	Care Climate Questionnaire (HCCQ)
Multidimensional scale of perceived social support (MSPSS)	[26,72,85,86,87,88]	Self-reported	Patients. Target women with diabetes and critical social support	Perceived social support	12 items including 3 subscales (family, friends, and significant others)	7-point Likert-type scale	Literature Review
Patient Assessment of Chronic Illness Care-Short Form (PACIC-SF)	[89,90,91,92]	Self-reported	Patients. Target primary care patients	Perceived self-management support	11 items Unidimensional scale	5-point Likert-type scale	Chronic Care Model (CCM)
Perceived social support from friends (PSS-Fr) and from family (PSS-Fa) Scales	[22,93]	Self-reported	Students. Target undergraduates	Perceived social support	20 items PSS-Fr Unidimensional scale 20 items PSS-Fa Unidimensional scale	Multichotomous response	Literature Review
Perceptions of Collaboration Questionnaire (PCQ)	[58,94]	Self-reported	Wife and husband dyad. Target married couples	Perceptions of collaboration (solving everyday problems and making decisions)	9 items including 3 subscales (cognitive compensation, interpersonal enjoyment, and frequency of collaboration)	5-point Likert scale	Literature Review
Scales to measure social support for diet and exercise behaviors	[23,93,95,96,97]	Self-reported	Students and staff. Target psychology students and staff members of a health-promotion research study	Perceived social support specific to health-related eating and exercise behaviors	10-item Social Support for Eating Habits (SSEH) scale: 2 subscales (encouragement; discouragement); 13-item Social Support for Physical Activity (SSPA) scale: 2 subscales (participation, rewards, and punishments)	5-point Likert scale	Literature review and structured in-depth interviews with people who were in the process of changing their diet and/or exercise habits report
Social Provision Scale (SPS)	[21,98,99]	Self-reported	College students and public-school teachers. Target different types of population	Social support	24 items including 6 subscales (attachment, reassurance of worth, reliable alliance, social integration, guidance, and opportunity for nurturance)	4-point Likert scale	Model of the Social Provisions

**Table 2:** Assessment results of each instrument development and content validity according to the COSMIN checklist.

Instrument	References	Instrument Development			Content Validity	Comments
		Design	Cognitive Interview	Total Instrument Development		
Brief Family Assessment Measure (Brief FAM-III)	[65]	A	A	A	A	
	[64]	-	-	-	-	Information about instrument development and content validity not reported
Chronic Illness Resources Survey (CIRS)	[67]	-	-	-	-	Information about instrument development and content validity not reported
	[66]					
Diabetes Caregiver Activity and Support Scale (D-CASS)	[37]	A	-	D	D	Important methodological flaws pilot test and content validity
Diabetes Care Profile (DCP)	[39]	A	-	D	-	Important methodological flaws in the, pilot test and content validity. This instrument evolved from the instrument Diabetes Educational Profile (DEP)
	[38]	-	-	-	-	Information about instrument development and content validity were not reported
	[7]					
	[40]					
	[41]					
	[42]					
Diabetes Family Behavior Checklist (DFBC)	[31]	A	A	A	D	Content validity: important methodological flaws in relevance and comprehensibility (professionals)
	[43]	-	-	-	-	Information about instrument development and content validity were not reported
	[48]					
	[45]					
	[46]					
	[47]					
	[44]					
Diabetes Family Behavior Checklist-II (DFBC-II)	[29]	-	-	-	-	Information about instrument development and content validity were not reported
	[51]					
	[49]					
	[50]					
	[52]					
Diabetes Family Support and Conflict scale (DFSC)	[53]	A	A	A	A	
	[54]	-	-	-	A	Information about instrument development were not reported
Diabetes Mellitus 2 treatment adherence scale version III (EATDM-III)	[55]	-	-	-	D	Information about instrument development were not reported. Not clear if professionals were asked about the relevance of the instrument’s items
Diabetes Support Scale (DSS)	[28] [42]	-	-	-	-	Information about instrument development and content validity were not reported
Empowerment questionnaire	[46]	-	-	-	-	This instrument comprised questions from other validated instruments. Information about instrument design, pilot test, and content validity were not reported
Family Adaptability and Cohesion Evaluation Scale (FACES IV)	[69]	-	-	-	-	This instrument was the latest version of the original FACES Information about instrument design, pilot test, and content validity were not reported
	[70]					
	[68]					
Family APGAR Index	[30]	-	-	-	-	Information about instrument development and content validity were not reported
	[32]					
	[73]					
	[74]					
	[72]					
	[71]					
	[15]					
Family Assessment Device (FAD)	[27]	-	-	-	-	Information about instrument development and content validity were not reported
	[75]					
	[76]					
	[16]					
	[77]					
Family-Carer Diabetes Management Self-Efficacy Scale (F-DMSES)	[56]	A	A	A	D	Content validity: small number of professionals involved in relevance evaluation
Family Emotional Involvement and Criticism Scale (FEICS)	[24]	A	D	D	D	Important methodological flaws in the design, pilot test, and content validity
	[78]	-	-	.	-	Information about instrument development and content validity were not reported
	[58]					
Family and Friend Involvement in Adults’ Diabetes (FIAD)	[93]	A	A	A	A	
	[58]	-	-	-	-	Information about instrument development and content validity were not reported
Family Function Questionnaire (FFQ)	[79]	A	A	A	D	Content validity: not clear about number of, or if, professionals were asked about relevance and comprehensiveness
	[17]	-	-	-	-	Information about instrument development and content validity were not reported
Family Functioning Style Scale	[81]	-	-	-	-	Information about instrument development and content validity were not reported
	[80]					
Family Support Scale adapted for African American women with type 2 Diabetes Mellitus (FSS-AA)	[59]	A	A	A	D	Content validity: the number of professionals involved in data analysis was not clear
Helping for Health Inventory: Couples Version (HHI-C)	[60]	-	-	-	-	Information about instrument development and content validity were not reported
Instrumental Expressive Social Support Scale (IESS)	[83]	-	-	-	-	Information about instrument development and content validity were not reported
	[82]					
Important Other Climate Questionnaire (IOCQ)	[84]	A	D	D	D	Important methodological flaws in the design, pilot test, and content validity.
	[58]	-	-	-	-	Information about instrument development and content validity were not reported
Multidimensional Diabetes Questionnaire (MDQ)	[25]	A	D	D	D	Cognitive interview: it was not clear if the patients were asked about comprehensibility and comprehensiveness; Content validity: it was not clear how many professionals were involved and if they were asked about the relevance and comprehensiveness of the items
	[61]	-	-	-	-	Information about instrument development and content validity were not reported
Multidimensional scale of perceived social support (MSPSS)	[26]	A	D	D	D	Cognitive interview: it was not clear if the patients were asked about comprehensibility and comprehensiveness; Content validity: it was not clear how many professionals were involved in data analysis and if they were asked about the relevance and comprehensiveness of the items
	[86]	A	D	D	D	
	[87]	-	-	-	-	Information about instrument development and content validity were not reported
	[72]					
	[85]					
	[88]					
Patient Assessment of Chronic Illness Care-Short Form (PACIC-SF)	[89]	A	-	D	-	No information about cognitive interview and about content validity
	[90]	-	-	-	-	Information about instrument development and content validity were not reported
	[91]					
	[92]					
Perceived social support from friends (PSS-Fr) and from family (PSS-Fa) Scales	[22]	A	-	D	-	No information about cognitive interview or about content validity
	[93]	-	-	-	-	Information about instrument development and content validity were not reported
Perceptions of Collaboration Questionnaire (PCQ)	[94]	A	-	D	-	No information about cognitive interview or about content validity
	[58]	-	-	-	-	Information about instrument development and content validity were not reported
Scales to measure social support for diet and exercise behaviors	[23]	A	D	D	D	Cognitive interview: it was not clear if the patients were asked about comprehensibility and comprehensiveness of the instrument; Content validity: it was not clear how many professionals were involved in data analysis and if they were asked about the relevance and comprehensiveness of the items
	[97]	A	A	A	A	
	[96]	-	-	-	-	Information about instrument development and content validity were not reported
	[93]					
	[95]					
Social Provision Scale (SPS)	[21]	A	D	D	D	Cognitive interview: it was not clear if patients were asked about the comprehensibility and comprehensiveness of the instrument; Content validity: it was not clear how many professionals were involved in data analysis and if they were asked about the relevance and comprehensiveness of the items
	[98]	-	-	-	-	Information about instrument development and content validity were not reported
	[99]					
Social support scale for self-care in middle-aged patients (S4-MAD)	[62]	A	A	A	A	
Unsupportive social interaction scale (USIS)	[63]	A	A	A	D	Content validity: it was not clear if the patients were asked about the relevance and comprehensiveness of the items

NOTE: “A”: Adequate; “D”: Doubtful; “-”: Information was not reported.

**Table 3:** Results of assessment of measurement properties according to COSMIN for each instrument.

		Methodological Result							
		Quality							
Instrument	References	Box 3	Box 4	Box 5	Box 6	Box 7	Box 8	Box 9	Box 10
		Structural Validity	Internal Consistency	Cross-Cultural Validity/Measurement Invariance	Reliability	Measurement Error	Criterion Validity	Hypothesis Testing for Construct Validity	Responsiveness
Brief FAM-III	[65]	I	V		-	-	-	-	-
	[64]	-	-	-	-	-	-	-	-
CIRS	[67]	-	V	-	V	-	-	A	
	[66]	-	I	-	-	-	-	-	-
D-CASS	[37]	A	V	-	V	-	-		-
DCP	[39]	V	V	-	-	-	V	A	
	[38]	-	V	-	-	-	-	-	-
	[7]	-	-	-	-	-	-	-	-
	[40]								
	[41]								
	[42]	-	V	-	-	-	-	-	-
DFBC	[31]	-	V	-	V	-	-	A	
	[43]	-	V	-	-	-	-	-	-
	[48]	-	V	-	-	-	-	-	-
	[45]	A	V	-	I	-		A	
	[46]	-	-	-	-	-	-	-	-
	[47]	-	V	-	-	-	-	-	-
	[44]	-	V	-	-	-	-	-	-
DFBC-II	[29]	-	V	-	V	-	-	A	-
	[51]	-	-	-	-	-	-	-	-
	[49]	-	V	-	-	-	-	-	-
	[50]	-	V	-	-	-	-	-	-
	[52]	-	-	-	-	-	-	-	-
DFSC	[53]	A	V	-	-	-	-	-	-
	[54]	A	V	-	D	-	-	-	-
EATDM-III	[55]	A	V	-	-	-	-	-	-
DSS	[28]	-	V	-	-	-	V	A	
	[42]	-	-	-	-	-	-	-	-
Empowerment questionnaire	[46]	A	V	-	I	-	-	D	-
FACES IV	[69]	V	V	-	-	-	V	-	-
	[70]	-	-	-	-	-	-	-	-
	[68]	-	V	-	-	-	-	-	-
Family APGAR Index	[30]	-	V	-	-	-	-	A	-
	[32]	-	V	-	V	-	-	A	-
	[73]	-	-	-	-	-	-	-	-
	[74]	-	-	-	-	-	-	-	-
	[72]	-	-	-	-	-	-	-	-
	[71]	-	-	-	-	-	-	-	-
	[15]	-	-	-	-	-	-	-	-
FAD	[27]	-	V	-	-	-	V	A	-
	[75]	-	V	-	-	-	-	-	-
	[76]	-	V	-	D	-	-	-	-
	[16]	-	V	-	-	-	-	-	-
	[77]	-	-	-	-	-	-	-	-
F-DMSES	[56]	A	V	-	V	-	-	-	D
FEICS	[24]	-	V	-	-	-	-	-	-
	[78]	V	V	-	-	-	V	A	-
	[58]	-	-	-	-	-	-	-	-
FIAD	[93]	V	V	-	V	-	V	A	-
	[58]	-	V	-	-	-	-	-	-
FFQ	[79]	-	V	-	I	-	-	-	-
	[17]	-	V	-	-	-	-	-	-
Family Functioning Style Scale	[81]	A	V	-	-	-	-	-	-
	[80]	-	-	-	-	-	-	-	-
FSS-AA	[59]	A	V	-	V	-	V	-	-
HHI-C	[60]	-	V	-	V	-	V	-	-
IESS	[83]	-	V	-	-	-	-	-	-
	[82]	V	V	-	-	-	-	A	-
IOCQ	[84]	V	V	-	V	-	-	A	-
	[58]	-	V	-	-	-	-	-	-
MDQ	[25]	V	V	-	D	-	-	A	-
	[61]	-	V	-	-	-	-	-	-
MSPSS	[26]	V	V	-	V	-	-	-	-
	[86]	A	V	-	-	-	V	A	-
	[87]	-	V	-	-	-	-	-	-
	[72]	-	-	-	-	-	-	-	-
	[85]	-	V	-	-	-	-	-	-
	[88]	-	-	-	-	-	-	-	-
PACIC-SF	[89]	A	V	-	-	-	-	A	-
	[90]	-	-	-	-	-	-	-	-
	[91]	-	-	-	-	-	-	-	-
	[92]	-	-	-	-	-	-	-	-
PSS-Fr and PSS-Fa	[22]	A	V	-	-	-	-	-	-
	[93]	-	V	-	-	-	-	-	-
PCQ	[94]	V	V	-	-	-	-	-	-
	[58]	-	V	-	-	-	-	-	-
Scales to measure social support for diet and exercise behaviors	[23]	A	V	-	D	-	V	A	-
	[97]	V	V	-	-	-	-	-	-
	[96]	-	V	-	-	-	-	-	-
	[93]	-	V	-	-	-	-	-	-
	[95]	-	V	-	-	-	-	-	-
SPS	[21]	V	V	-	-	-	-	A	-
	[98]	-	V	-	-	-	-	-	-
	[99]	-	V	-	-	-	-	-	-
S4-MAD	[62]	V	V	-	V	-	-	-	-
USIS	[63]	A	V	-	A	-	-	A	-

Note: Brief FAM-III: Brief Family Assessment Measure-Brief; CIRS: Chronic Illness Resources Survey; D-CASS: Diabetes Caregiver Activity and Support Scale; DCP: Diabetes Care Profile; DFBC: Diabetes Family Behavior Checklist; DFBC-II: Diabetes Family Behavior Checklist-II; DFSC: Diabetes Family Support and Conflict scale; EATDM-III: Diabetes Mellitus 2 treatment adherence scale version III; DSS: Diabetes Support Scale; FACES IV: Family Adaptability and Cohesion Evaluation Scale; FAD: Family Assessment Device; F-DMSES: Family-Carer Diabetes Management Self- Efficacy Scale; FEICS: Family Emotional Involvement and Criticism Scale; FIAD: Family and Friend Involvement in Adults’ Diabetes; FFQ: Family Function Questionnaire; FSS-AA: Family Support Scale adapted for African American women with type 2 Diabetes Mellitus; HHI-C: Helping for Health Inventory: Couples Version; IESS: Instrumental Expressive Social Support Scale; IOCQ: Important Other Climate Questionnaire; MDQ: Multidimensional Diabetes Questionnaire; MSPSS: Multidimensional scale of perceived social support; PACIC-SF: Patient Assessment of Chronic Illness Care-Short Form; PSS-Fr: Perceived social support from friends; PSS-Fa: Perceived social support from family; PCQ: Perceptions of Collaboration Questionnaire; SPS: Social Provision Scale; S4-MAD: Social support scale for self-care in middle-aged patients; USIS: Unsupportive social interaction scale. “V”: Very Good; “A”: Adequate; “D”: Doubtful; “I”: Inadequate; “-”: Information was not reported.

**Table 4:** Rating scores of measurement properties for each instrument.

Instrument	References	Rating Scores of Measurement Properties							
		Structural Validity	Internal Consistency	Cross-Cultural Validity/Measurement Invariance	Reliability	Measurement Error	Criterion Validity	Hypothesis Testing for Construct Validity	Responsiveness
Brief FAM-III	[65]	−	+	NR	NR	NR	NR	NR	NR
	[64]	NR	NR	NR	NR	NR	NR	NR	NR
CIRS	[67]	NR	−	NR	+	NR	NR	?	NR
	[66]	NR	−	NR	NR	NR	NR	NR	NR
D-CASS	[37]	−	+	NR	+	NR	NR	NR	NR
DCP	[39]	?	−	NR	NR	NR	−	+	NR
	[38]	NR	+	NR	NR	NR	NR	NR	NR
	[7]	NR	NR	NR	NR	NR	NR	NR	NR
	[40]	NR	NR	NR	NR	NR	NR	NR	NR
	[41]	NR	+	NR	NR	NR	NR	NR	NR
	[42]	NR	NR	NR	NR	NR	NR	NR	NR
DFBC	[31]	NR	−	NR	−	NR	NR	−	NR
	[43]	NR	−	NR	NR	NR	NR	NR	NR
	[48]	NR	+	NR	NR	NR	NR	NR	NR
	[45]	?	+	NR	+	NR	NR	NR	NR
	[46]	NR	NR	NR	NR	NR	NR	NR	NR
	[47]	NR	+	NR	NR	NR	NR	NR	NR
	[44]	NR	+	NR	NR	NR	NR	NR	NR
DFBC-II	[29]	NR	−	NR	?	NR	NR	NR	NR
	[51]	NR	NR	NR	NR	NR	NR	NR	NR
	[49]	NR	+	NR	NR	NR	NR	NR	NR
	[50]	NR	+	NR	NR	NR	NR	NR	NR
	[52]	NR	NR	NR	NR	NR	NR	NR	NR
DFSC	[53]	−	+	NR	NR	NR	NR	NR	NR
	[54]	+	+	NR	?	NR	NR	NR	NR
EATDM-III	[55]	-	+	NR	NR	NR	NR	NR	NR
DSS	[28]	NR	+	NR	NR	NR	−	−	NR
	[42]	NR	NR	NR	NR	NR	NR	NR	NR
Empowerment questionnaire	[46]	?	+	NR	−	NR	NR	?	NR
FACES IV	[69]	+	+	NR	NR	NR	+	+	NR
	[70]	NR	NR	NR	NR	NR	NR	NR	NR
	[68]	NR	+	NR	NR	NR	NR	NR	NR
Family APGAR Index	[30]	NR	−	NR	NR	NR	NR	+	NR
	[32]	NR	+	NR	+	NR	NR	NR	NR
	[73]	NR	NR	NR	NR	NR	NR	NR	NR
	[74]	NR	NR	NR	NR	NR	NR	NR	NR
	[72]	NR	NR	NR	NR	NR	NR	NR	NR
	[71]	NR	NR	NR	NR	NR	NR	NR	NR
	[15]	NR	NR	NR	NR	NR	NR	NR	NR
FAD	[27]	NR	+	NR	NR	NR	−	NR	NR
	[75]	NR	+	NR	NR	NR	NR	NR	NR
	[76]	NR	−	NR	?	NR	NR	NR	NR
	[16]	NR	+	NR	NR	NR	NR	NR	NR
	[77]	NR	NR	NR	NR	NR	NR	NR	NR
F-DMSES	[56]	−	+	NR	−	NR	NR	NR	−
FEICS	[24]	NR	+	NR	NR	NR	NR	NR	NR
	[78]	−	+	NR	NR	NR	−	+	NR
	[58]	NR	NR	NR	NR	NR	NR	NR	NR
FIAD	[93]	+	−	NR	−	NR	−	+	NR
	[58]	NR	+	NR	NR	NR	NR	NR	NR
FFQ	[79]	NR	−	NR	−	NR	NR	+	NR
	[17]	NR	+	NR	NR	NR	NR	NR	NR
Family Functioning Style Scale	[81]	-	+	NR	NR	NR	NR	NR	NR
	[80]	NR	NR	NR	NR	NR	NR	NR	NR
FSS-AA	[59]	−	+	NR	−	NR	−	−	NR
HHI-C	[60]	NR	−	NR	?	NR	−	−	NR
IESS	[83]	NR	+	NR	NR	NR	NR	NR	NR
	[82]	−	+	NR	NR	NR	NR	NR	NR
IOCQ	[84]	+	+	NR	−	NR	NR	−	NR
	[58]	NR	+	NR	NR	NR	NR	NR	NR
MDQ	[25]	+	+	NR	?	NR	NR	NR	NR
	[61]	NR	+	NR	NR	NR	NR	NR	NR
MSPSS	[26]	−	+	NR	?	NR	NR	NR	NR
	[86]	−	+	NR	NR	NR	−	+	NR
	[87]	NR	+	NR	NR	NR	NR	NR	NR
	[72]	NR	NR	NR	NR	NR	NR	NR	NR
	[85]	NR	+	NR	NR	NR	NR	NR	NR
	[88]	NR	NR	NR	NR	NR	NR	NR	NR
PACIC-SF	[89]	−	+	NR	NR	NR	NR	+	NR
	[90]	NR	NR	NR	NR	NR	NR	NR	NR
	[91]	NR	NR	NR	NR	NR	NR	NR	NR
	[92]	NR	NR	NR	NR	NR	NR	NR	NR
PSS-Fr and PSS-Fa	[22]	?	+	NR	NR	NR	NR	NR	NR
	[93]	NR	+	NR	NR	NR	NR	NR	NR
PCQ	[94]	−	−	NR	NR	NR	NR	NR	NR
	[58]	NR	−	NR	NR	NR	NR	NR	NR
Scales to measure social support for diet and exercise behaviors	[23]	−	−	NR	?	NR	−	−	NR
	[97]	+	+	NR	NR	NR	NR	NR	NR
	[96]	NR	+	NR	NR	NR	NR	NR	NR
	[93]	NR	+	NR	NR	NR	NR	NR	NR
	[95]	NR	−	NR	NR	NR	NR	NR	NR
SPS	[21]	−	−	NR	NR	NR	NR	+	NR
	[98]	NR	−	NR	NR	NR	NR	NR	NR
	[99]	NR	−	NR	NR	NR	NR	NR	NR
S4-MAD	[62]	+	+	NR	+	NR	NR	NR	NR
USIS	[63]	−	+	NR	?	NR	NR	NR	NR

Note: “+” = sufficient; “−“= insufficient, “?” = indeterminate; NR = measurement was not reported. Brief FAM-III: Brief Family Assessment Measure-Brief; CIRS: Chronic Illness Resources Survey; D-CASS: Diabetes Caregiver Activity and Support Scale; DCP: Diabetes Care Profile; DFBC: Diabetes Family Behavior Checklist; DFBC-II: Diabetes Family Behavior Checklist-II; DFSC: Diabetes Family Support and Conflict scale; EATDM-III: Diabetes Mellitus 2 treatment adherence scale version III; DSS: Diabetes Support Scale; FACES IV: Family Adaptability and Cohesion Evaluation Scale; FAD: Family Assessment Device; F-DMSES: Family-Carer Diabetes Management Self-Efficacy Scale; FEICS: Family Emotional Involvement and Criticism Scale; FIAD: Family and Friend Involvement in Adults’ Diabetes; FFQ: Family Function Questionnaire; FSS-AA: Family Support Scale adapted for African American women with type 2 Diabetes Mellitus; HHI-C: Helping for Health Inventory: Couples Version; IESS: Instrumental Expressive Social Support Scale; IOCQ: Important Other Climate Questionnaire; MDQ: Multidimensional Diabetes Questionnaire; MSPSS: Multidimensional scale of perceived social support; PACIC-SF: Patient Assessment of Chronic Illness Care-Short Form; PSS-Fr: Perceived social support from friends; PSS-Fa: Perceived social support from family; PCQ: Perceptions of Collaboration Questionnaire; SPS: Social Provision Scale; S4-MAD: Social support scale for self-care in middle-aged patients; USIS: Unsupportive social interaction scale.

## Data Availability

The data that support the findings of this study are available from the corresponding author upon reasonable request.

## Supplementary Materials

The following supporting information can be downloaded at: https://www.mdpi.com/article/10.3390/ijerph20021325/s1, Table S1: Search strategy used for Medline; Table S2: Summary information of each included study in chronological order; Table S3: Summary of results for measurement properties for each instrument.
