# Supplementary material for: Diabetes Mellitus Family Assessment Instruments: A Systematic Review of Measurement Properties
Source: Int J Environ Res Public Health. 2023 Jan 11;20(2):1325. doi: 10.3390/ijerph20021325 (PMC9859216; doi:10.3390/ijerph20021325)
Supplement: Supplementary file 1 [file ijerph-20-01325-s001.zip › Supplementary File S1. Search strategy used for Medline.pdf]

Table S1. Search strategy used for Medline

Search	Query	Results
**S1**	diabetes OR diabet* OR "diabetes mellitus"	825,255
**S2**	family OR famil* OR peers OR friend* OR neighbour* OR "fellow patient*" OR colleague* OR "internet contact*" OR "pen friend*"	1,804,198
**S3**	"family assessment instruments" OR "family management measure" OR "family evaluation instrument" OR "diabetes family conflict scale" OR "dfcs" OR "diabetes family responsibility questionnaire" OR "dfrq" OR "dfrc" OR "family summary of diabetes self care activities" OR "fsdca" OR "family illness perception questionnaire diabetes version" OR "diabetes family behavior scale" OR "dfbs" OR "diabetes behavior checklist" OR "dbc" OR "family illness perception questionnaire" OR "diabetes social support questionnaire family version" OR "DSSQ family version" OR "diabetes family behavior checklist" OR "dfbc" OR "ipq diabetes version" OR "assumption of diabetes management scale"	12,710
**S4**	"family relation*" OR "social support" OR "family function" OR "family dynamic*"	112,885
**S5**	"outcomes measure*" OR "cross cultural validity" OR responsiveness OR validity OR reliability	4,033,309
**S6**	S1 AND S2 AND S3 AND S4 AND S5	25
**S7**	Limiters: English; Portuguese; Spanish; French, from 2010	18

Note: "*" to include all derivatives of that word or concept.
