# Supplementary material for: Diabetes Mellitus Family Assessment Instruments: A Systematic Review of Measurement Properties
Source: Int J Environ Res Public Health. 2023 Jan 11;20(2):1325. doi: 10.3390/ijerph20021325 (PMC9859216; doi:10.3390/ijerph20021325)
Supplement: Supplementary file 1 [file ijerph-20-01325-s001.zip › Supplementary File S2. Summary information of each included study.pdf]

Table S2. Summary information of each included study in chronological order

Reference	Instrument	Study Design	Sample characteristics		Distribution of score Mean (± SD)	Setting	Concept of family
			n	Mean Age (years)			
Good et al., 1979	Family APGAR Index	Quantitative: Cross-sectional	58	Nonclinical group:<br>28.3 (Females)<br>28.6 (Males)<br>Clinical group:<br>32.0 (Females)<br>33.0 (Males).	Nonclinical: 8.2<br>Clinical: 5.9	Community sector and mental health center	Psychosocial group, including the patient and one or more persons in which there is a commitment for members to care for each other
Smilkstein et al., 1982	Family APGAR Index cross-cultural potential for the Family APGAR	Quantitative: Cross-sectional	1395[a]	19.7 (Study 1)	Study 1: 7.6 (±2.3)<br>Study 2: 7.6 (±2.3)<br>Study 3: 7.6 (±2.4)<br>Study 4: 8.2 (±2.1)<br>Study 5: 5.8 (±2.7)	Community sector and health care centers	NR
Epstein et al., 1983	Family Assessment Device (FAD)	Quantitative	503	NR	FAD subscales[b]<br>PS: 2.3 (± 0. 5)<br>CM: 2.3 (± 0.5)<br>R: 2.4 (± 0.4)<br>AR: 2.4 (± 0.6)<br>AI: 2.2 (± 0.5)<br>BC: 2.0 (± 0.4)<br>GF: 2.2 (± 0.6)	University and psychiatric hospital	NR
Procidano & Heller, 1983	Perceived social support from friends (PSS-Fr) and from family (PSS-Fa) Scales	Quantitative	432[c]	19.0 (Study 1 and study 2)<br>20.5 (Study 3)	NR	University	NR
Schafer et al., 1986	Diabetes Family Behavior Checklist (DFBC)	Quantitative: Longitudinal	68	35.6 (Adult group)<br>14.6 (Adolescents group)	DFBC positive score initial<br>- Adults: 19.2 (±5.6)	Health care centers	Closest family members (in terms of day-to day contact)

					- Adolescents 24.3 (± 6.0)<br>DFBC Negative score initial<br>- Adults: 13.7 (±3.6)<br>- Adolescents: 15.9 (±5.0)<br>DFBC 6 months Positive score<br>- Adults: 20.5 (±5,6)<br>- Adolescents: 24.0 (±5.9)<br>DFBC 6 months Negative score<br>- Adults: 12.7 (±3.2)<br>- Adolescents: 15.8 (±5.7)		
Cutrona & Russell,1987	Social Provision Scale (SPS)	Quantitative	1972	NR	82.5 (±9.9)	Community settings	NR
Sallis et al., 1987	Scales to measure social support for diet and exercise behaviors	Quantitative	211[d]	36.0 (Study I)<br>21.4 (Study II)	NR	Community settings	Family was defined as "members of the household"
Glasgow & Toobert, 1988	Diabetes Family Behavior Checklist-II (DFBC-II)	Quantitative: Longitudinal	127	60.8	DFBC-II positive score: 2.2 (±0.6)<br>DFBC-II negative score: 1.9 (±0.6)	Community settings	Partner or significant other persons
Zimet et al., 1988	Multidimensional Scale of Perceived Social Support (MSPSS)	Quantitative: Cross-sectional	275	18.6	5.8 (±0.9)	University	NR
Shields et al., 1992	Family Emotional Involvement and Criticism Scale (FEICS)	Quantitative: Cross-sectional	83	55.5	NR	University medical care	Family is more than marital status or people who share a house

Fitzgerald et al., 1996	Diabetes Care Profile (DCP)	Quantitative	792[e]	61.0 (Study 1)<br>54.0 (Study 2)	NR	Community settings and university medical center	NR
Talbot et al., 1997	Multidimensional Diabetes Questionnaire (MDQ)	Quantitative: Cross-sectional	249	54.9	MQD scales<br>Interference :1.6 (±1.5)<br>Severity: 3.6 (±1.6)<br>Support: 4.1 (±1.5)<br>Positive reinforcing behaviors: 2.6 (±1.9)<br>Misguided support behaviors:1.4 (±1.5)<br>Self-efficacy: 64.2 (±20.8)<br>Outcome expectancies: 88.5 (±13.0)	Diabetes Education Centers	NR
Glasgow et al., 1999	Diabetes Support Scale (DSS)	Quantitative: Cross-sectional	221	45.1	4.6 (±1.4)	Diabetes internet news groups and list servers	NR
Larraín et al., 2003	Family Functioning Style Scale	Quantitative: Cross-sectional	264	NR	NR	Community settings	NR
Glasgow et al., 2005	Chronic Illness Resources Survey (CIRS)	Quantitative	293	60.7 (Usual care group)<br>61.1 [Intervention group (IG)]	CIRS (Pre intervention): 2.7 (±0.6)<br>CIRS (12-month follow-up): 2.8 (± 0.5)	Primary care clinics	NR
Williams et al., 2006	Important Other Climate Questionnaire (IOCQ)	Quantitative: longitudinal	865	64.5	Important Others Climate Questionnaire (diet):<br>Baseline: 4.7(±1.4)<br>1 month: 4.8 (±1.7)	Community setting	Person selected as being important can be a friend or coworker
Roncone et al., 2007	Family Function Questionnaire (FFQ)	Quantitative	92	52.1	NR	Day-Hospital	NR
Olson et al., 2010	Family Emotional Involvement and Criticism Scale (FEICS)	Quantitative: Cross-sectional	87	55.0	Family´s Perceived Criticism (PC): 6.6<br>Intensity of Emotional Involvement (EI): 16.6	University-based ambulatory medical clinic	Focused in patients' marital or committed relationship

Note: NR: Not Reported; a) *n*= 1395 (Study 1 *n*= 529; Study 2 *n*= 486; Study 3 *n*= 297; Study 4; *n*= 133; Study 5 *n*=158); b) FAD subscales - CM: Communication; GF: General functioning; PS: Problem solving; R: Roles; AR: Affective responsiveness; AI: Affective involvement; BC: Behavior Control; c) *n*= 432 (Study 1 *n*= 222; study 2 *n*= 105; study 3 *n*= 105); d) *n*= 211 (Study 1 *n*= 40; Study 2 *n*= 171); e) *n*= 792 (Study 1 *n*= 440; Study 2 *n*= 352); f) *n*= 380 (Qualitative sample *n*= 38; Exploratory Factor Analysis (EFA) *n*= 204; Confirmatory Factor Analysis (CFA) *n*= 138); g) *n*= 904 (Study 1 *n*= 392; study 2 *n*= 512); h) Intervention group (IG); i) Control group (CG)

Rosland et al., 2010	Family APGAR Index	Quantitative: Cross-sectional	578	63.0	NR	Primary health care centers university	NR
Toro et al., 2010	Family APGAR Index	Quantitative: Association	118	NR	NR	Medical center	NR
Paddison, 2010	Diabetes Family Support and Conflict scale (DFSC)	Quantitative: Cross-sectional	629	63.0	NR	Primary health care	Person considered by patient as part of his/her immediate or extended family
Berg et al., 2011	Perceptions of Collaboration Questionnaire (PCQ)	Quantitative: Cross-sectional	300	43.9 (Middle-aged couples_ wife)<br>45.8 (Middle-aged couples_ man)<br>62.2 (Older couples_ wife)<br>64.7 (Older couples_ man)	NR	Community settings	NR
Palma et al., 2011	Diabetes Family Behaviour Checklist (DFBC)	Quantitative: Cross-sectional	48	35.5	DFBC positive: 17.1 (±5.7)<br>DFBC negative:12.9 (±5.2)	Community center and medical center	NR
García-Huidobro et al., 2011	Family Functioning Style Scale	Quantitative: Controlled clinical trial	243	53.4 (IG)<br>53.5 CG1<br>56.2 (CG2)	Baseline<br>IG: 92.9 (±12.0);<br>CG1: 93.7 (±9.5)<br>CG2: 92.7 (±11.6)<br>12 months<br>IG: 94.9 (±13.8)<br>CG1: 93.1 (±18.7)<br>CG2: 97.1 (±9.8)	Primary care clinic	NR
Noroozi et al., 2011	Social Support Scale for Exercise Behavior	Quantitative: Cross-sectional	348	50.0	Social support friend subscale: 2.5 ± 1.0<br>Social support family subscale: 2.5± 0.9	Diabetic institute	NR
Olson, 2011	Family Adaptability and Cohesion Evaluation Scale IV (FACES IV)	Quantitative: Cross-sectional	469	28.0	NR	Community settings	NR
Karlsen et al., 2012	Diabetes Family Behavior Checklist (DFBC)	Quantitative: Cross-sectional	425	58.1	DFBC positive: 2.5 (± 0.7)<br>DFBC negative: 1.8 (± 0.7)	Community settings	Close relatives or others living with

							the diagnosed person
Goetz et al., 2012	Patient Assessment of Chronic Illness Care-Short Form (PACIC-SF)	Quantitative: observational	264	64.0	NR	University hospital	NR
Naderimagham et al., 2012	Social support scale for self-care in middle-aged patients with type II diabetes (S4-MAD)	Quantitative: Cross-sectional	380[e]	45.7 (Qualitative sample)<br>50.6 (EFA sample)<br>50.8 (CFA sample)	2.4 (± 1.5)	University of Medical Sciences: diabetes screening centers	NR
Park et al., 2012	Multidimensional Scale of Perceived Social Support (MSPSS)	Quantitative: Cross-sectional	123	53.4	Family source: 5.3 (± 1.5)<br>Spouse source: 4.9(±1.8)<br>Friends source 4.1(±1.9)	Community settings	NR
Hara et al., 2013	Diabetes Family Behavior Checklist (DFBC)	Quantitative: Cross-sectional	327	61.9 (Insulin group)<br>65.3 (Oral hypoglycemic agents' group)	NR	University hospitals, core hospitals, medical centers and clinics	Family members are who are living with the patients
Takenaka et al., 2013	Family Adaptability and Cohesion Evaluation Scale IV-16 (FACESKG IV)	Quantitative: Cross-sectional	121	52.1	NR	Diabetic clinic	Family members are who live with patients or immediate relatives
Bahremand, et al., 2014	Family Assessment Device (FAD)	Quantitative: Descriptive, correlational	225	NR	132.6 (±126.1)	Diabetic care centers	NR
Chesla et al., 2014	Diabetes Care Profile (DCP)	Quantitative: Quasi-experimental	178	59.4 (Women)<br>62.8 (Man)	Family instrumental support:<br>Woman 3.96 (±0.9)<br>Men 3.90 (±0.8)<br>Family emotional support:<br>Woman: 3.9 (±0.7)<br>Men 4.0 (± 0.7)	Community settings	NR
Hara et al., 2014	Empowerment questionnaire for patients with type 2 diabetes	Quantitative	338	64.1 (Man)<br>63.0 (Women)	NR	University hospitals, core hospitals, medical centers, and clinics	NR
He at al., 2014	Family Assessment Device (FAD)	Quantitative: Cross-sectional	261	54.4 (People with depressive symptoms)	FAD subscales [g] (People with diabetes with depressive symptoms)	Hospital	NR

				53.6 (People without depressive symptoms)	PS: 2.1 (±0.1)<br>CM: 2.2 (±0.3)<br>R: 2.4 (±0.1)<br>AR: 2.1 (±0.4)<br>AI: 2.4 (± 0.2)<br>BC: 2.1 (± 0.1)<br>GF: 1.9 (± 0.2)<br>FAD subscales (People with diabetes without depressive symptoms):<br>PS: 1.9 (± 0.3)<br>CM: 2.0 (± 0.4)<br>R: 2.1 (± 0.4)<br>AR: 2.1 (± 0.4)<br>AI: 2.2 (± 0.4)<br>BC: 2.1(± 0.3)<br>GF: 1.8 (± 0.4)		
Karlsen & Bru, 2014	Diabetes Family Behavior Checklist (DFBC)	Quantitative: Prospective longitudinal	296	59.0	DFBC Scores (Respondents participating in T1 and T2):<br>DFBC positive: 2.7(±0.8)<br>DFBC negative: 1.8 (± 0.7)<br>DFBC Scores (Respondents not participating in T2):<br>DFBC positive: 2.6 (±0.8)<br>DFBC negative: 1.9 (± 0.8)	Community settings	NR
Mayberry & Osborn, 2014	Diabetes Family Behaviour Checklist- II (DFBC-II)	Quantitative: Cross-sectional	192	51.6	DFBC-II Supportive behaviors: 2.4 (±1.0)	Health centers	NR
Mayberry et al., 2014	Diabetes Family Behaviour Checklist- II (DFBC-II)	Quantitative: Cross-sectional	183	51.2	DFBC-II scores<br>Supportive behaviors: 2.4 (± 1.0)<br>Obstructive behaviors: 2.1 (± 0.9)	Health care centers	NR

Pereira et al., 2014	Multidimensional Diabetes Questionnaire (MDQ)	Quantitative	387	59.0	NR	Health care centers	NR
Senol-Durak., 2014	Scale of perceived social support (MSPSS)	Quantitative	455	50.9	NR	Diabetes' hospital and health care centers	NR
Baron-epel et al., 2015	Unsupportive social interaction scale (USIS)	Quantitative: cross-sectional	764	61.3	USIS subscales<br>Interference: 1.9 (±0.9)<br>Insensitivity: 2.1 (± 0.8)	Health care centers	NR
DePalma et al., 2015	Diabetes Family Behavior Checklist (DFBC)	Quantative	119	65.8	DFBC positive: 17.9 (±6.9)<br>DFBC negative:12.1(± 4.8)	Community setting	NR
Heiss & Petosa, 2015	Scales to measure social support for diet and exercise behaviors	Quantitative: cross-sectional	181	53.3	NR	Community setting	NR
Littlewood et al., 2015	Family support scale adapted for African American women with type 2 diabetes mellitus (FSS-AA)	Quantitative: randomized controlled trial	200	52.0 (IG)<br>54.0 (CG)	IG: 1.9 (± 1.2)<br>CG: 2.3 (± 1.2)	Primary care and community setting	NR
Mayberry et al., 2015	Diabetes Family Behavior Checklist- II (DFBC-II)	Quantitative: cross-sectional	192	51.6	DFBC-II scores<br>Supportive behaviors: 2.4 (± 1.0)<br>Obstructive behaviors: 2.1 (± 1.0)	Health center	NR
Odume et al., 2015	Family APGAR Index<br>Multidimensional Scale of Perceived Social Support (MSPSS)<br>-	Quantitative: cross-sectional	145	50.0	APGAR class<br>High family functioning: 64%<br>Moderately dysfunctional family: 33%<br>Severely dysfunctional family: 2.8%<br>MSPSS score<br>High acuity: 57.9%<br>Moderate acuity: 35.2%<br>Low acuity: 6.9%	National hospital	NR

Úrzua et al., 2015	Diabetes Mellitus 2 treatment adherence scale version III (EATDM-III)	Quantitative: cross-sectional	247	58.7	NR	Primary care clinics or private consultations	NR
Azmoude et al., 2016	Family Assessment Device (FAD)	Quantitative: cross-sectional	180	35.0	NR	Health care centers	NR
Batty & Fain, 2016	Family APGAR Index	Quantitative: descriptive, correlational	77	44.0	8.0 (± 2.4)	Hospital and private medical practice	NR
Bhandari & Kim, 2016	Multidimensional Scale of Perceived Social Support (MSPSS)	Quantitative: mixed method	230	56.9	5.7 (± 1.0)	Private clinic and hospital	NR
Cai & Hu, 2016	Chronic Illness Resources Survey (CIRS)	Quantitative: Quasi-experimental	54	65.3 (People with diabetes)<br>63.4 (Patient's family members)	5.8 (± 1.7)	Community settings and community health center	Whoever the patients consider them to be
Chun et al., 2016	Social Provision Scale (SPS)	Quantitative: cross-sectional	162	61.0	3.1 (± 0.5)	Community settings	NR
Nicolucci et al., 2016	Patient Assessment of Chronic Illness Care-DAWN Short Form (PACIC-DSF)	Quantitative: multinational, interdisciplinary and multistakeholder	8596	54.0	NR	Community settings	NR
Shawon et al., 2016	Diabetes Care Profile (DCP)	Quantitative: cross-sectional	144	54.4	Social and family support Scores<br>Support needed scale: 4.2 (± 0.7)<br>Support received scale: 3.7 (± 0.9)<br>Support attitude scale: 4.1 (± 0.9)	Public hospital	NR
Vallis et al., 2016	Patient Assessment of Chronic Illness Care-DAWN Short Form (PACIC-DSF)	Quantitative: multinational, interdisciplinary and multistakeholder	500	54.0	35.3 (± 24.4)	Community settings	NR

Mar-García et al., 2017	Family APGAR Index	Quantitative: Transversal and correlational	100	57.7	NR	Health centers	NR
Ramkisson et al., 2017	Diabetes Care Profile (DCP)	Quantitative: cross-sectional	401	53.7	NR	Public health facilities and private medical practices	NR
Regufe, 2017	Instrumental Expressive Social Support Scale (IESS)	Quantitative: observational, descriptive and transversal	144	53.6	3.7 (±0.8)	Public hospital	NR
Scarton et al., 2017	Diabetes Caregiver Activity and Support Scale (D-CASS)	Quantitative: cross-sectional	101	51.0	44.1 (±12.3)	Hospital and community settings	Family caregivers are defined as any relative, partner, or friend who provides assistance and support to a person with diabetes
Sofulu et al., 2017	Diabetes Family Support and Conflict Scale	Quantitative: cross-sectional	158	59.1	29.5 (±3.8)	University hospital	NR
Tanaka et al., 2017	Helping for Health Inventory - Couples Version (HHI-C)	Quantitative: cross-sectional	268	56.8	Baseline HHI-C: 33.6 (±10.7)	Community settings	NR
Yerusalem et al., 2017	Scale of perceived social support (MSPSS)	Quantitative: correlational	60	34.2	NR	Community settings	NR
Harper et al., 2018	Diabetes Family Behavior Checklist-II (DFBC-II)	Quantitative: exploratory	53	57.3	DFBC-II supportive harmful score: 2.6 (±0,9)	Health centers	NR
Iloh et al., 2018	Family Assessment Device (FAD)	Quantitative: Clinic-based descriptive	120	36.8	NR	Tertiary hospital	NR
Lima et al., 2018	Instrumental Expressive Social Support Scale (IESS)	Quantitative: cross-sectional	964	74.4	NR	Community-dwelling	NR
Shamali et al., 2018	Brief Family Assessment Measure (Brief FAM-III)	Quantitative: cross-sectional	58	79.3	Brief FAM-III scales scores General Scale: 53.8 (±10.8) Self-Rating Scale: 51.4 (±11.2)	University hospital	NR

					Dyadic Relationships Scale: 51.5 (±8.5)		
Spencer et al., 2018	Diabetes Support Scale (DSS)	Quantitative: cohort	222	48.9	NR	Health center	NR
Vallis et al., 2018	Patient Assessment of Chronic Illness Care-DAWN Short Form (PACIC-DSF)	Quantitative: multinational survey	1368[d]	25.0 (Emerging adults)<br>47.7 (Adults)	Emerging adults score: 47.4 (±22.5)	Community settings	NR
Wichit et al., 2018	Family- Carer Diabetes Management Self- Efficacy Scale (F-DMSES)	Quantitative: cross-sectional	70	NR	Baseline score: 50.2 (±11.0)	Community setting	Considered persons living with individuals with T2DM in same residency; being a spouse, child, grandchild, sibling or friend of individuals with T2DM
Bennich et al., 2019	Brief Family Assessment Measure (Brief FAM-III)	Quantitative: explorative, descriptive cross-sectional	127	65.3	NR	Hospital, University and general practitioners	NR
Mayberry et al., 2019	Family and Friend Involvement in Adults' Diabetes (FIAD)	Quantitative: mixed-methods (cross-sectional observational study and longitudinal randomized controlled trial	904[f]	57.5 (study 1)<br>56.0 (study 2)	Study 1<br>Helpful involvement score: 1.9 (±0.9)<br>Harmful involvement score: 1.6 (±0.6)<br>Study 2<br>Helpful involvement score: 2.0 (±0.9)<br>Harmful involvement score: 1.7 (±0.6)	University Medical Center and Primary Care clinics	Not only those who live together are considered family
McEwen et al., 2019	Scales to measure social support for diet and exercise behaviors<br>Perceived social support from friends (PSS-Fr) and	Quantitative: experimental repeated-measures	314	53.5 (patients)<br>47.3 (family members)	NR	Community settings and clinics	NR

	from family (PSS-Fa) Scales						
Shuhaida et al., 2019	Diabetes Care Profile (DCP)	Quantitative: cross-sectional	338	60.9	NR	Health clinic	NR
Andrade et al., 2020	Family APGAR Index	Quantitative: cross-sectional	236	71.6	NR	Basic Health Units	NR
Basinger, 2020	Family Adaptability and Cohesion Evaluation Scale (FACES IV)	Quantitative	159	58.9	Balanced Cohesion Subscale Score: 4.0 (±1.0)	Hospital and University	NR
Horikawa et al., 2020	Diabetes Family Behaviour Checklist (DFBC)	Quantitative: cross-sectional	289	62.0	NR	Health clinics	NR
Pamungkas & Chamroonsawasdi, 2020	Family Function Questionnaire (FFQ)	Quantitative: Quasi-experimental	60	56.2 (IG)<br>54.5 (CG)	NR	Community health center	NR
Yeung et al., 2020	Social Provision Scale (SPS)	Quantitative: cohort study	907	65.8 (Diabetes & Multiple Chronic Illnesses group)<br>63.6 (Without disease)	Diabetes & Multiple Chronic Illnesses group score: 69.5 (±9.3)<br>Without disease score: 73.4 (±9.6)	Community settings	NR
Al-Ghafri et al., 2021	Scales to measure social support for diet and exercise behaviors	Quantitative: randomized controlled trial	227	44.2	IG Family Social Support scores<br>Baseline 13.5 (± 4.5)<br>12 months 16.1 (± 4.5)<br>IG Friends Social Support scores:<br>Baseline 9.5 (± 2.8)<br>12 months 12.2 (± 5.1)<br>Comparison group Family Social Support scores:<br>Baseline 10.5 (± 3.5)<br>12 months 12.0 (± 5.3)<br>Comparison group Friends Social Support scores:<br>Baseline 9.2 (± 3.1);	Primary health centers	NR

					12 months 9.6 (± 3.0)		
Mayberry et al., 2021	Family Emotional Involvement and Criticism Scale (FEICS)<br>- Family/Friend Involvement in Adults' Diabetes (FIAD)<br>- Perceptions of Collaboration Questionnaire (PCQ)<br>- Important Other Climate Questionnaire	Quantitative	379	59.1	NR	University Medical center	Family is more than marital status or people who share a house
