# Supplementary material for: Diabetes Mellitus Family Assessment Instruments: A Systematic Review of Measurement Properties
Source: Int J Environ Res Public Health. 2023 Jan 11;20(2):1325. doi: 10.3390/ijerph20021325 (PMC9859216; doi:10.3390/ijerph20021325)
Supplement: Supplementary file 1 [file ijerph-20-01325-s001.zip › Supplementary File S3. Summary of results for measurement properties for each instrument.pdf]

Table S3. Summary of results for measurement properties for each instrument

Instrument	Reference	Status of instrument	Country/ Language	Measurement properties	
				**Reliability**	**Validity**
Brief Family Assessment Measure-Brief (Brief FAM-III)	Shamali et al., 2018	Translation and validation study	Denmark (Danish)	**Internal consistency:**<br>Total scale (α): 0.94<br>General Scale (α): 0.83<br>Self-Rating Scale (α): 0.84<br>Dyadic Relationships scale(α): 0.90	**Structural validity:**<br>CFA was done for three-factor model. *p*< 0.0001 (correlations between the three factors)
	Bennich et al., 2019	Translation	Denmark (Danish)	No value	No value
Chronic Illness Resources Survey (CIRS)	Glasgow et al., 2005	Original	USA (English)	**Internal consistency:**<br>Total scale(α): 0.82<br>Subscales (α): ranged from 0.40 ("Media and policy") to 0.86 ("Physician/health care team")<br>**Test-retest:**<br>r= 0.70 for total scale<br>r= ranged 0.55 to 0.66	**Convergent validity**<br>r≥ 0.40, *p*< 0.05 (CIRS scales and other support measures)
	Cai & Hu, 2016	Translation	China (Chinese)	**Internal consistency:**<br>Total scale (α): 0.87	No value
Diabetes Caregiver Activity and Support Scale (D-CASS)	Scarton et al., 2017	Original	USA (English)	**Internal consistency:**<br>Total scale (α): 0.82<br>**Test-retest:**<br>ICC: 0.70	**Content validity:**<br>I-CVIs: 0.86 to 1.0<br>Scale I-CVI: 1.0<br>**Structural validity:**<br>EFA was done. 1-factor solution supported the unidimensional scale<br>**Criterion- related validity:**<br>r= 0.65, *p*< 0.01 (D-CASS total with single-item criterion measuring)
Diabetes Care Profile (DCP)	Fitzgerald et al., 1996	Original	USA (English)	**Internal consistency:**<br>DCP scales (α:): ranged from 0.60 to 0.95 (community study)	**Structural validity:**<br>CFA and EFA were done.<br>**Concurrent validity**

		and validation study		Total scale (α): ranged from 0.66 to 0.94 (medical center study)	r= 0.32, *p*≤ 0.01 (DCP positive attitude scale and Social provisions scale)<br>r= 0.32, *p*≤ 0.01 (DCP understanding of management practice scale and Social provisions scale)<br>r= 0.51, *p*≤ 0.01 (DCP support attitudes scale and Social provisions scale)<br>r= - 0.34, *p*≤ 0.01 (DCP negative attitude scale and Social provisions scale)<br>r= - 0.33 *p*≤ 0.01 (DCP social and personal factors scale and Social provisions scale)<br>r= 0.34, *p*≤ 0.01 (DCP control problems scale and CES scale)<br>r= 0.48, *p*≤ 0.01 (DCP social and personal factors scale and CES scale)<br>r=0.48, *p*≤ 0.01 (DCP negative attitude scale and CES scale)<br>r= 0.32, *p*≤ 0.01 (DCP monitoring barriers and CES scale)<br>r= - 0.53, *p*≤ 0.01 (DCP positive attitude scale and CES scale)<br>r= - 0.42, *p*≤ 0.01 (DCP self-care ability scale and CES scale)<br>r= - 0.35, *p*≤ 0.01 (DCP self-care adherence scale and CES scale)<br>r= -0.35, *p*≤ 0.01 (DCP support attitudes scale and CES)<br>r= 0.32, *p*≤ 0.01 (DCP positive attitude scale and Happiness and satisfaction scale)<br>r= 0.30, *p*≤ 0.01 (DCP self-care ability scale and Happiness and satisfaction scale)<br>**Construct validity:**<br>Community Study:

					p= 0.05 (DCP control problems scale comparison between the three patients' groups). Patients with NIDDM no insulin reported fewer control problems<br>p= 0.05 (DCP understanding management practice scale comparison between the three patients' groups). Patients with IDDM reported the best understanding of their sell-care<br>p= 0.05 (DCP Social and personal factors scale comparison between patients using insulin and patients with NIDDM not using insulin. Patients not using insulin reported less impact in their social and personal life<br>p= 0.05 (DCP positive attitude scale comparison between patients NIDDM using insulin and patients NIDDM not using insulin). Patients not using insulin reported more positive outlook<br>p= 0.05 (DCP medical barriers scale comparison between patients IDDM and patients with NIDDM. Patients with NIDDM have more difficulty in following their insulin regimen.<br>p= 0.05 (DCP monitoring barriers scale comparison between patients with NIDDM using insulin and NIDDM not using insulin and patients with IDDM). Patients with NIDDM using insulin reported the fewest problems with monitoring

					r≥ 0.20, p≤0.01 (DCP scales: self-care ability, self-care adherence and control problems with glycosylated hemoglobin<br>Medical Center study:<br>*p*= 0.05 (DCP control problems scale, medical barriers scale and understanding management practice scale comparison between patients with IDDM and patients with NIDDM). Patients with IDDM reported more control problems, more difficulty following their insulin regimen and a better understanding of their self-care.<br>*p*= 0.05 (DCP social and personal factors scale comparison between patients using insulin with patients with NIDDM not using insulin. Diabetes had less impact on the social and personal lives of patients with NIDDM not using insulin.
	Chesla et al., 2014	Translation	USA (Chinese)	**Internal consistency**<br>Total scale (α): 0.70	No value
	Ramkisson et al., 2017	Original	South Africa (English)	No value	No value
	Spencer et al., 2018	Original	USA (English)	No value	No value
	Shawon et al., 2016	Translation	Bangladesh (Bengali)	No value	No value
	Shuhaida et al., 2019	Translation	Malaysia (Malay)	**Internal consistency**<br>Total scale (α): 0.76	No value
Diabetes Family Behavior Checklist (DFBC)	Schafer et al, 1986	Original	USA (English)	**Internal consistency:**<br>Adults and family DFBC positive score (α): 0.73	**Convergent validity:**

		and validation study		Adults and family DFBC negative score (α): 0.43<br>Adolescents and family positive score (α): 0.63<br>Adolescents and family negative score (α): 0.60<br>**Test-retest:**<br>r= 0.58 to 0.72, $p \leq 0.001$ (adults and family)<br>r= 0.60 and 0.75, $p \leq 0.05$ (adolescents and family for positive score)<br>r=0.60 to 0.28 (adolescents and family for negative scores)	r= ranged from 0.27 $p < 0.10$, to 0.68, $p < 0.001$ (adults and family positive and negative DFBC scores)<br>r= ranged from 0.01 to 0.27; all NS (adolescents and family DFBC scores)<br><br>**Concurrent validity**<br>r< 0.30; NS (adults DFBC scores and adherence measures)<br>r= - 0.43 and -0.48; $p < 0.09$ (adolescents initial DFBC scores and adherence measures – dietary exchange deviations at the 6-mo follow-up
	DePalma et al., 2011	Original	USA (English)	**Internal consistency**<br>DFBC – positive score (α): 0.69<br>DFBC – negative score (α): 0.72	No value
	Karlsen et al., 2012	Original	Norway (English)	**Internal consistency**<br>DFBC – positive score (α): 0.79<br>DFBC – negative score (α): 0.78	No value
	Hara et al., 2013	Translation and validation study	Japan (Japanese)	**Internal consistency**<br>Insulin group DFBC positive score (α): 0.95<br>Insulin group DFBC negative score (α): 0.93<br>Oral hypoglycemic agents' group positive score (α): 0.95<br>Oral hypoglycemic agents' group negative score (α): 0.94<br>**Test-retest:**<br>ICC= 0.89	**Structural validity:**<br>Factor analysis was done. Identified two subscales (''Positive feedback'' and ''Negative feedback'') except on three questions<br>**External validity:**<br>r= 0.24, $p < 0.01$ (Insulin group DFBC positive score with questionnaire on self-managed dietary behavior)<br>r= 0.29, $p < 0.001$ (Insulin group DFBC positive score with questionnaire on self-managed exercise behavior)<br>r= 0.24, $p < 0.05$ (Oral hypoglycemic agents' group DFBC positive score with questionnaire on self-managed exercise behavior)

					r= 0.30, *p*< 0.001 (Insulin group DFBC positive score with ADS subscale Sense of self-control)<br>r= 0.32, *p*< 0.001 (Oral hypoglycemic agents' group DFBC positive score with ADS subscale Sense of self-control)<br>r= 0.22, *p*< 0.01 (Insulin group DFBC positive score with ADS subscale Efforts for symptom management)<br>r= 0.19, *p*< 0.05 (Insulin group DFBC negative score with ADS subscale Efforts for symptom management)<br>r= 0.17, *p*< 0.05 (Oral hypoglycemic agents' group DFBC positive score with ADS subscale Efforts for symptom management)<br>r= 0.16, *p*< 0.05 (Oral hypoglycemic agents' group DFBC positive score with ADS subscale Efforts for symptom management)<br>r= - 0.20, *p*< 0.05 (Oral hypoglycemic agents' group DFBC negative score with questionnaire on self-managed dietary behavior)<br>r= - 0.18, *p*< 0.05 (Insulin group DFBC negative score with questionnaire on self-managed exercise behavior)
	Hara et al., 2014	Translation	Japan (Japanese)	No value	No value
	Karlsen & Bru, 2014	Original	Normay (English)	**Internal consistency**<br>DFBC – positive score (α): 0.79<br>DFBC – negative score (α): 0.78	No value
	DePalma et al., 2015	Original	USA (English)	**Internal consistency**<br>DFBC – positive score(α): 0.78<br>DFBC – negative score (α): 0.74	No value

Diabetes Family Behavior Checklist-II (DFBC-II)	Glasgow & Toobert, 1988	Original	USA (English)	**Internal consistency**<br>DFBC II – positive score(α): 0.71<br>DFBC II – negative score (α): 0.64<br>**Test-retest:**<br>r= ranged from 0.55 to 0.70 (DFBC II –positive score and DFBC II – negative score)<br>r= 0,59 (DFBC-II diet scale)<br>r= 0,76 (DFBC-II exercise scale)<br>r= 0,22 (DFBC-II glucose testing)<br>r= 0,05 (DFBC-II medication support)	**Convergent validity:**<br>r= 0.53, $p<0.001$ (adults and family positive DFBC II score)<br>r= 0.56, $p<0.001$ (adults and family negative DFBC II score)<br>r= ranged from 0.40 to 0.56 (patient and family versions of DFBC II subscales, except glucose testing).<br>**Predictive validity**:<br>Regimen-component-specific measures of family support were stronger predictors of regimen adherence than were the overall positive and negative summary scores
	Mayberry et al., 2014	Original and translation	USA (English and Spanish)	No value	No value
	Mayberry & Osborn, 2014	Original and translation	USA (English and Spanish)	**Internal consistency**<br>DFBC II – positive score (α): 0.85<br>DFBC II – negative score (α): 0.78	No value
	Mayberry et al., 2015	Original and translation	USA (English and Spanish	**Internal consistency**<br>DFBC II – positive score (α): 0.85<br>DFBC II – negative score (α): 0.78	No value
	Harper et al., 2018	Original	USA (English)	No value	No value
Diabetes Family Support and Conflict scale (DFSC)	Paddison, 2010	Original and validation	New Zealand (English)	**Internal consistency**<br>Family support scale (α): 0.84<br>Family conflict scale (α): 0.75	**Structural validity:**<br>EFA was done. Identified the presence of two factors (family support and family conflict)
	Sofulu et al., 2017	Translation and validation	Torque (Turkish)	**Internal consistency**<br>Total scale (α): 0.74<br>Family support subscale (α): 0.77<br>Family conflict subscale (α): 0.92<br>r= 0.82 (Family support subscale and Family conflict subscale)	**Content validity:**<br>Scale I-CVI: 0.15<br>**Structural validity:**<br>Factor analysis was done. Identified the presence of two factors (family support and family conflict)

				r= 0.79 (Family support subscale and DFSC total)<br>r= 0.64 (Family conflict subscale and DFSC total)	
Diabetes Mellitus 2 treatment adherence scale version III (EATDM-III)	Úrzua et al., 2015	Original and validation	Chile (Spanish)	**Internal consistency**<br>Total scale(α): 0,87<br>Family support subscale (α) = 0.84<br>Physical exercise subscale (α) = 0.88<br>Medical control and treatment subscale (α)= 0.79<br>Community support and organization subscale (α) = 0.79<br>Information subscale (α) = 0.72<br>Diet subscale (α) = 0.57<br>r= ranged from 0.23 to 0.55 (item-total)<br>r= ranged from 0.35 to 0.83 (item-dimension)	**Structural validity:**<br>EFA was done. Identified the presence of six factors
Diabetes Support Scale (DSS)	Glasgow et al., 1999	Original	USA (English)	**Internal consistency**<br>Total scale(α): 0.93	**Concurrent validity:**<br>r= ranged from 0.26 to 0.45 (between DSS, ISES and CISS)<br>**Construct validity:**<br>r= ranged from 0.84 to 0.89 (three subscales of DSS)<br>r= 0.26, p< 0.001 (D-Net use: number of logins and DSS)<br>r= 0.25, p< 0.01 (DSS and foot care)<br>r= - 0.26, p< 0.01 (DSS and Illness intrusiveness)
	Spencer et al., 2018	Original	USA (English)	No value	No value
Empowerment questionnaire	Hara et al., 2014	Original and validation	Japan (Japanese)	**Internal consistency**<br>Five scale(α): ranged from 0.70 to 0.89<br>**Test-retest:**	**Structural validity:**<br>Principal components analysis was done

				r= ranged from 0.56 to 0.76	r= ranged from 0.41 to 0.85, except for the question A7 (each item)<br>**Concurrent validity:**<br>Comparison of scale scores between the non-treatment and the four treatment groups Psychological impact and glycemic control: Psychological impact is lower in patients with good glycemic control $p < 0.028$, data do not show
Family Adaptability and Cohesion Evaluation Scale (FACES IV)	Olson, 2011	Original and validation	USA (English)	**Internal consistency**<br>Enmeshed scale (α): 0.77<br>Disengaged scale (α):0.87<br>Balanced Cohesion scale (α):0.89<br>Chaotic scale (α): 0.86<br>Balanced Flexibility scale (α): 0.84<br>Rigid scale (α): 0.82	**Concurrent validity:**<br>r= 0.98 (FACES IV Balanced cohesion scale and SFI)<br>r= - 0.90 (FACES IV Disengaged scale and SFI)<br>r= - 0.71 (FACES IV Chaotic scale and SFI)<br>r= 0.99 (FACES IV Balanced flexibility scale and SFI)<br>r= 0.89 (FACES IV Balanced cohesion scale and FSS)<br>r= - 0.84 (FACES IV Disengaged scale and FSS)<br>r= - 0.67 (FACES IV Chaotic scale and SFI)<br>r= 0.91 (FACES IV Balanced flexibility scale and SFI)<br>r= 0.95 (FACES IV Balanced cohesion scale and FSS)<br>r= - 0.93 (FACES IV Disengaged scale and FSS)<br>r= - 0.71 (FACES IV Chaotic scale and SFI)<br>r= 0.95 (FACES IV Balanced flexibility scale and SFI)<br>**Structural validity:**

					EFA and CFA were done. Identified the presence of six factors<br>r= - 0.68 (balanced cohesion and Chaotic scale)<br>r= - 0.11(balanced cohesion and Rigid scale)<br>r= 0.82 (balanced flexibility and Disengaged scale)<br>r= - 0.09 (balanced flexibility and Enmeshed scale)
	Takenaka et al., 2013	Translation (Used FACESKG Japanese version)	Japan (Japanese)	No value	No value
	Basinger, 2020	Original	USA (English)	**Internal consistency:**<br>Balanced Cohesion Subscale (α): 0.95	No value
Family APGAR Index	Good et al., 1979	Original and validation study	USA (English)	**Internal consistency:**<br>r= ranged from 0.24 to 0.67 (inter-item)<br>r= 0.93 (scores on items one, three, and five and items two and four)	**Construct validity**<br>t=3.43, p< 0.001 (Clinical and nonclinical group)<br>r= 0.80 (Family APGAR Index and Plessy-Satterwhite score)<br>r= 0.64 (Family APGAR Index and therapists' family evaluation score for clinical group)
	Smilkstein et al., 1982	Original	USA (English)	**Internal consistency:**<br>Family APGAR Index Subscale (α): 0.80 (study 1)<br>Three-Choice Format Family APGAR Index (α): 0.80 (study 2)<br>Five-Choice Format Family APGAR Index (α): 0.86 (study 2)<br>**Test-retest reliability:**	**Construct validity:**<br>t= 3.15, p< 0.01 (Family APGAR score and Friends APGAR score)<br>t= 11.65, p< 0.001 (Well-Adjusted and Maladjusted student Groups)

				Family APGAR Index (α): 0.83 (National Taiwan University Study)	
	Rosland et al., 2010	Original	USA (English)	No value	No value
	Toro & Garcés, 2010	Translation	Chile (Spanish)	No value	No value
	Odume et al, 2015	Translation	Nigeria (English)	No value	No value
	Batty & Fain, 2016	Original	USA (English)	Not value	No value
	Andrade et al., 2020	Translation	Brazil (Portuguese)	No value	No value
Family Assessment Device (FAD)	Epstein et al., 1983	Original	USA (English)	**Internal consistency**<br>Problem solving scale (α): 0.74<br>Communication scale (α): 0.75<br>Roles scale (α): 0.72<br>Affective responsiveness scale (α): 0.83<br>Affective involvement scale (α): 0.78<br>Behavior control scale (α): 0.72<br>General functioning scale (α): 0.92	**Construct validity:**<br>F= 15.51, $p< 0.0001$ (Problem solving scale: clinical group and nonclinical group)<br>F= 15.30, $p< 0.0001$ (Communication scale: clinical group and nonclinical group)<br>F= 30.25, $p< 0.0001$ (Roles scale: clinical group and nonclinical group)<br>F= 7.03, $p< 0.01$ (Affective responsiveness scale: clinical group and nonclinical group)<br>F= 10.07, $p< 0.01$ (Affective involvement scale: clinical group and nonclinical group)<br>F= 5.90, $p< 0.02$ (Behavior control scale: clinical group and nonclinical group)<br>F= 25.00, $p< 0.0001$ (General functioning scale: clinical group and nonclinical group)<br>**Predictive validity:**

					FAD predicted 22% (r= 0.47) of the variance in the Morale score for husbands and 17% (r= 0.34 for wives)<br>**Concurrent validity:**<br>Regression analysis was done. FAD predicted 28%, r= 0.53 of the variance on the Locke Wallace Marital Satisfaction Scale (husbands and wives analyzed separately)
	Bahremand et al., 2014	Translation	Iran (Persian)	**Internal consistency**<br>Total scale (α): 0.91	No value
	He at al., 2014	Translation	China (Chinese)	**Internal consistency**<br>Total scale (α): ranged from 0.53 to 0.94<br>**Test-retest reliability:**<br>r= ranged from 0.53 to 0.81	No value
	Azmoude et al., 2016	Translation	Iran (Persian)	**Internal consistency**<br>Total scale (α): 0.82	No value
	Iloh et al., 2018	Translation	Nigeria (English)	No value	No value
Family-Carer Diabetes Management Self- Efficacy Scale (F-DMSES)	Wichit et al., 2018	Translation and validation	Thailand (Thai)	**Internal consistency**<br>Total scale (α): 0.89<br>General diet and blood glucose monitoring subscale(α): 0.85<br>Medication and complication subscale (α): 0.87<br>Diet in different situations subscale (α): 0.83<br>Weight control and physical activities subscale (α): 0.70<br>r (inter-item): 0.41<br>**Test-retest reliability:**<br>ICC= 0.56<br>r= 0.74, $p< 0.001$	**Content validity:**<br>CVI: 0.93<br>**Structural validity:**<br>EFA was done. Identified the presence of four factors<br>**Changes overtime:**<br>effect size =0.9, $p< 0.001$ (F- DMSES at baseline and after intervention)
	Olson et al., 2010	Original	USA (English)	**Internal consistency**	No value

Family Emotional Involvement and Criticism Scale (FEICS)				Perceived Criticism subscale (α): 0.82<br>Emotional Involvement subscale (α): 0.74	
	Shields et al., 1992	Original and validation	USA (English)	**Internal consistency**<br>PC subscale (α): 0.82<br>EI subscale (α): 0.74	**Structural validity:**<br>CFA was done. Identified the presence of two factors<br>**Construct validity:**<br>r= 0.59, p< 0.001 (EI subscale and FACES III Cohesion subscale)<br>r= 0.50, p< 0.001 (EI subscale and FACES III Adapt. subscale)<br>r= - 0.35, p< 0.001 (EI subscale and ISEL Tangible subscale)<br>r= -0.34, p< 0.001 (EI subscale and ISEL Belonging subscale)<br>r= - 0.39, p< 0.001 (EI subscale and ISEL Appraisal subscale)<br>r= - 0.44, p< 0.001 (PC subscale and FACES III Cohesion subscale)<br>r= - 0.37, p< 0.001 (PC subscale and FACES III Adapt. subscale)<br>r= 0.30, p< 0.001 (PC subscale and ISEL Tangible subscale)<br>r= 0.35, p< 0.001 (PC subscale and ISEL Belonging subscale)<br>r= 0.25, p< 0.001 (PC subscale and ISEL Appraisal subscale)<br>**Criterion validity:**<br>r= 0.38, p≤ 0.001 (PC subscale and Depression)<br>r= 0.35, p≤ 0.001 (PC subscale and Anxiety)<br>r= 0.25, p≤ 0.05 (EI subscale and Depression)<br>r= 0.22, p≤ 0.05 ((EI subscale and Anxiety)

	Mayberry et al., 2021	Original	USA (English)	No value	No value
Family and Friend Involvement in Adults' Diabetes (FIAD)	Mayberry et al., 2019	Original and validation	USA (English)	**Internal consistency**<br>Helpful involvement (α): 0,86 (study 1)<br>Harmful involvement (α): 0.72 (study 1)<br>Helpful involvement (α): 0.87 (study 2)<br>Harmful involvement (α): 0.63 (study 2)<br>**Test-retest reliability:**<br>ICC= 0.65 (helpful)<br>ICC= 0.60 (harmful)<br>r= 0.64, $p< 0.001$ (helpful)<br>r= 0.61, $p< 0.001$ (harmful)	**Structural validity:**<br>EFA and CFA were done. Identified the presence of two factors<br>r=0.40 and 0.44, $p< 0.001$ (FIAD helpful and harmful scores)<br>**Construct validity:**<br>r=ranged from 0.35 to 0.36, $p< 0.001$ (FIAD helpful subscale and PCQ subscales)<br>r= 0.36, $p< 0.001$ (FIAD helpful subscale and IOCQ scale)<br>r= 0.14, $p< 0.001$ (FIAD harmful subscale and PCQ Cognitive Compensation)<br>r= - 0.12, $p< 0.001$ (FIAD harmful subscale and IOCQ scale)<br>**Criterion validity:**<br>r= 0.15, $p= 0.002$ (FIAD helpful subscale and PDSMS)<br>r= - 0.25, $p< 0.001$ (FIAD harmful subscale and PDSMS)<br>r= -0.18, $p= 0.001$ (FIAD harmful subscale and PDQ problem eating behavior subscale)<br>r= 0.32, $p< 0.001$ (FIAD harmful subscale and PDQ problem eating behavior subscale)<br>r= 0.31, $p< 0.001$ (FIAD helpful subscale and PDQ Use of Dietary Information for Decision Making subscale)

					r= -0.20, *p*< 0.001 (FIAD harmful subscale and PDQ Use of Dietary Information for Decision Making subscale)<br>r= 0.20, *p*< 0.001 (FIAD helpful subscale and SDSCA Blood Glucose Testing subscale)<br>r= - 0.15, *p*< 0.001 (FIAD harmful subscale and SDSCA Blood Glucose Testing subscale)<br>r= 0.18 *p*= 0.001 (FIAD helpful subscale SDSCA Medication Adherence subscale)<br>r= - 0.26 *p*< 0.001(FIAD harmful subscale SDSCA Medication Adherence subscale)<br>**Predictive validity:**<br>β= 0.17, *p*= 0.011 (helpful involvement and independently predicted increased blood glucose testing from enrollment to three-month follow-up)<br>β= 0.12, *p*= 0.046 (harmful involvement independently predicted worsening HbA1c from enrollment to three-month follow-up**)**
	Mayberry et al., 2021	Original	USA (English)	**Internal consistency**<br>Helpful involvement (α): 0.87<br>Harmful involvement (α): 0.72	No value
Family Function Questionnaire (FFQ)	Roncone et al., 2007	Original and validation	Italy (Italian)	**Internal consistency:**<br>Total scale (α): 0.84<br>Problem solving subscale (α): 0.83<br>Communication skills subscale (α): 0.71<br>Personal goals subscale (α): 0.66<br>**Test-retest reliability:**<br>ICC: ranged from 0.60 to 0.74	**Construct validity:**<br>r= 0.73 to 0.57 (Problem solving subscale and their items)<br>r= 0.71 to 0.43 (Communication skills subscale and their items)<br>r= 0.68 to 0.40 (Personal goals subscale and their items)

					r= 0.72 (Problem Solving and Communication subscales)<br>r= 0.51 (Problem Solving and Personal Goal)<br>r= 0.46 (Communication and Personal Goals)<br>**Convergent validity:**<br>r= 0.31, *p*< 0.01 (FFQ and SF-36 subscale Vitality)<br>r=0.32, *p*< 0.01 (FFQ and SF-36 subscale Mental Health)<br>r= - 0.26, *p*< 0.05 (FFQ and PF Questionnaire Objective burden)<br>r= - 0.27, *p*< 0.05 (FFQ and PF Questionnaire Objective burden)
	Pamungkas & Chamroonsawasdi, 2020	Translation	Indonesia (Indonesian)	**Internal consistency:**<br>Total scale (α): 0.80	No value
Family Functioning Style Scale	Larraín et al., 2003	Translation and validation	Chile (Spanish)	**Internal consistency:**<br>Total scale (α): 0.79	**Structural validity:**<br>EFA was done. Identified the presence of three factors
	García-Huidobro et al., 2011	Validation	Chile (Spanish)	No value	No value
Family Support Scale adapted for African American women with type 2 diabetes mellitus (FSS-AA)	Littlewood et al., 2015	Original and validation	USA (English)	**Internal consistency:**<br>Total scale (α): 0.90<br>Parent and spouse/partner Support subscale (α): 0.86<br>Community and Medical Support subscale (α): 0.83<br>Extended Family & Friends Support subscale (α) 0.83<br>**Test-retest reliability:**	**Structural validity:**<br>EFA was done. Identified the presence of three factors<br>**Concurrent validity:**<br>r= 0.16, *p*< 0.05 (FSS-AA subscale parent and spouse/partner support and self-care)

				r= 0.69	r= 0.44, *p*< 0.025 (FSS-AA subscale community and medical support and self-care)<br>r= 0.30, *p*< 0.025 (FSS-AA subscale extended family and friends support and self-care)<br>r= 0.17, *p*< 0.05 (FSS-AA subscale community and medical support and empowerment)<br>r= 0.21, *p*< 0.025 (FSS-AA subscale extended family and friends support and empowerment)<br>r= 0.20, *p*< 0.025 (FSS-AA subscale extended family and friends support and self-efficacy)<br>r= - 0.20, *p*< 0.025 (FSS-AA subscale extended family and friends support and depression)<br>r= -0.22, *p*< 0.025 (FSS-AA subscale community and medical support and diabetes distress scale)<br>r= -0.27, *p*< 0.025 (FSS-AA subscale extended family and friends support and diabetes distress)<br>r= ranged from -0.40 to -014, *p*< 0.025 and p <0.05 (FSS-AA and DDS)
Helping for Health Inventory: Couples Version (HHI-C)	Tanaka et al., 2017	Original	Canada (English)	**Internal consistency:**<br>Total scale (α): 0.86<br>Conflict/Blame subscale (α): 0.89<br>Partner Investment subscale (α): 0.68<br>Resistance subscale (α): 0.66<br>**Test-retest reliability:**<br>Total scale (α): ranged from 0.83 to 0.87 (four time points)	**Structural validity:**<br>EFA was done. Identified the presence of three factors<br>**Convergent validity:**<br>r= 0.14, *p*= 0.03 (HHI-C and conflict engagement)<br>r= 0.15, *p*= 0.02 (HHI-C and compliance conflict resolution strategies)

				r= ranged from 0.69 to 0.80 (HHI-C across all four time points within the diabetes education group)	r= −0.13, *p*= 0.04 (HHI-C and positive problem-solving strategies)<br>r= 0. 27, *p*< 0.01 (HHI-C- Baseline and Diabetes Distress Scale)<br>r= 0.16, *p*= 0.01(HHI-C- Baseline and Patient Health Questionnaire)
Instrumental Expressive Social Support Scale (IESS)	Regufe, 2017	Translation	Portugal (Portuguese)	**Internal consistency:**<br>Total scale (α): 0.94<br>Familiar and socio-affective support subscale (α): 0.89<br>Sense of control subscale (α): 0.77<br>Financial support subscale (α): 0.85	No value
	Lima et al., 2018	Translation and validation	Portugal (Portuguese)	**Internal consistency:**<br>Total scale (α): 0.94<br>Familiar and socio-affective support subscale (α): 0.78<br>Sense of control subscale (α): 0.90<br>Financial support subscale (α): 0.80	**Structural validity:**<br>EFA and CFA were done. Identified the presence of three factors<br>**Convergent validity:**<br>r= 0.32, *p*= 0.000 (total score of social support and adherence)<br>r= 0.22, *p*= 0.000 (total score of social support and positive affect)<br>**Divergent validity:**<br>r= -0.37, *p*= 0.000 (total score of social support and negative affect)
Important Another Climate Questionnaire (IOCQ)	Williams et al., 2006	Original and validation	USA (English)	**Internal consistency:**<br>IOCQ–S scale (α): ranged from 0.87 to 0.90 (across all three time points)<br>IOCQ-D scale (α): 0.95 (across all three time points)<br>r (IOCQ–S item to total) = ranged from 0.59 to 0.73 (baseline) from 0.54 to 0.75 (one month) from 0.66 to 0.80 (6 months)<br>r (IOCQ–D item to total) = ranged from 0.75 to 0.87 (baseline), from 0.75 to 0.89 (one month), from 0.76 to 0.86 (6 months)	**Structural validity:**<br>CFA was done. Confirmed single-factor structure of the IOCQ in both its versions<br>**Construct validity (IOCQ-D):**<br>r= 0.23, *p*< 0.01 (IOCQ-D and 6-month autonomy for diet)<br>r= 0.22, *p*< 0.01 (IOCQ-D and competence for diet)<br>r= - 0.17, *p*< 0.05 (IOCQ-D and 6-month total calories)<br>r= - 0.32, *p*< 0.01 (IOCQ-D and percent calories from fat)

				**Test-retest reliability:**<br>r= 0.57 (IOCQ–D baseline to 1 month)<br>r= 0.53 (IOCQ–S baseline to 1 month)	r= - 0.30, $p < 0.001$ (IOCQ-D and saturated fat)<br>**Predictive validity (IOCQ-D):**<br>F (1, 15) = 3.28, $p < 0.08$, β=0.12 (baseline autonomous motivation for diet from baseline to six months)<br>**Predictive validity (IOCQ-S):**<br>F (1, 38) = 6.31, β= 0.10, $p < 0.05$ (one month predicted six-month levels of both autonomous motivation for cessation) and perceived competence, F (1, 38) =7.15, β= 0.13, $p < 0.01$, as well as autonomous motivation for taking medications, F (1, 372) = 4.27, β= 0.09, $p < 0.05$
	Mayberry et al., 2021	Original	USA (English)	**Internal consistency:**<br>IOCQ scale (α): 0.89	No value
Multidimensional Diabetes Questionnaire (MDQ)	Talbot et al., 1997	Original and validation	Canada (English)	**Internal consistency:**<br>MQD scales (α): ranged from 0.70 to 0.91<br>r= ranged from 0.01 to 0.68 (among MDQ scales)	**Structural validity:**<br>CFA was done. Confirmed three factor model for section I, two factor model for section II and two factor model for section III<br>**Construct validity:**<br>*p* values= ranged from 0.006 to 0.013 (MDQ scales and duration of diabetes, HbA1c and diabetes complications)<br>$p < 0.008$ and $p < 0.013$ (MQD scales and Behavioral - diet and exercise)<br>$p < 0.008$ and $p < 0.013$ (MQD scales and BDI-SF scale)<br>$p < 0.008$ and $p < 0.013$ (MQD scales and IHLC scale)

	Pereira et al., 2014	Translation	Portugal (Portuguese)	**Internal consistency:**<br>Section II positive support (α): 0.85<br>Section II negative support (α): 0.78	
Multidimensional scale of perceived social support (MSPSS)	Zimet et al., 1988	Original and validation	USA (English)	**Internal consistency:**<br>Total scale (α): 0.88<br>Significant other subscale (α): 0.91<br>Family subscale (α): 0.87<br>Friends subscale (α): 0.85<br>**Test-retest reliability:**<br>Total scale (α): 0.85<br>Significant other subscale (α): 0.72<br>Family subscale (α): 0.85<br>Friends subscale (α): 0.75	**Structural validity:**<br>CFA was done. Confirmed three subscales<br>**Divergent validity:**<br>r= - 0.24, $p< 0.01$ (MSPSS subscale family and depression)<br>r= - 0.18, $p< 0.01$(MSPSS subscale family and anxiety)<br>r= - 0.13, $p< 0.05$ (MSPSS subscale significant other and depression)<br>r= - 0.25, $p< 0.01$ (MSPSS total scale and depression)
	Park et al., 2012	Translation and validation	Republic of Korea (Korean)	**Internal consistency:**<br>Total scale (α): 0.90<br>Significant other subscale (α):0.87<br>Family subscale (α): 0.83<br>Friends subscale (α): 0.93<br>r= 0.53 to 0.69 (item-to-total scale)	**Structural validity:**<br>Factor analysis was done. Three factors were extracted<br>r= 0.36 to 0.55 (among factors)<br>**Concurrent validity:**<br>r= 0.66, $p< 0.001$ (MSPSS and PRQ)<br>**Convergent validity:**<br>r=0.26, $p< 0.001$ (MSPSS and Self-efficacy for Diabetes Scale<br>**Divergent validity:**<br>r= - 0,41, $p< 0.001$ (MSPSS and CES-D)
	Senol-Durak, 2014	Translation	Turkey (Turkish)	**Internal consistency:**<br>Significant other subscale (α): 0.96<br>Family subscale (α): 0.94<br>Friends subscale (α): 0.93	No value
	Odume et al, 2015	Original	Nigeria (English)	No value	No value
	Bhandari & Kim,2016	Translation	Nepal (Nepali)	**Internal consistency:**<br>Total scale (α): 0.88	No value

	Yerusalem et al., 2017	Translation	Greece (Greek)	No value	No value
Patient Assessment of Chronic Illness Care-Short Form (PACIC-SF)	Goetz et al., 2012	Translation and validation	Germany (German)	**Internal consistency:** Total scale (α): 0.87	**Structural validity:** Factor analysis was done. Was revealed one-dimensional structure **Convergent validity:** r= 0.82, p< 0.001 (PACIC short form and the 20-item PACIC mean score)
	Nicolucci et al., 2016	Original and translation	Multinational (17 countries)	No value	No value
	Vallis et al., 2016	Translation	Canada (English)	No value	No value
	Vallis et al., 2018	Original and Translation	Multinational	No value	No value
Perceived social support from friends (PSS-Fr) and from family (PSS-Fa) Scales	Procidano & Heller, 1983	Original and validation study	USA (English)	**Internal consistency:** Study 1- PSS-Fr (α): 0.88 Study 1- PSS- Fa (α): 0.90	**Structural validity:** Factor analysis was done. Was revealed that each scale was composed of a single factor **Divergent validity:** r= - 0.27, p< 0.01 (PSS-Fr and Langner symptom scores) r= - 0.29, p< 0.01 (PSS-Fa and Langner symptom scores)
	McEwen et al., 2019	Original and translation	USA (English and Spanish)	**Internal consistency:** PSS participants (α): 0.90 PSS family (α): 0.89	No value
Perceptions of Collaboration Questionnaire (PCQ)	Berg et al., 2011	Original	USA (English)	**Internal consistency:** Wife Cognitive Compensation subscale (α): 0.57 Husband Cognitive Compensation subscale (α): 0.61 Wife Interpersonal Enjoyment subscale (α): 0.59	**Structural validity:** CFA was done. Were established the three-factor structure

				Husband Interpersonal Enjoyment subscale (α): 0.65<br>Wife Frequency of Collaboration subscale (α): 0.80<br>Husband Frequency of Collaboration subscale (α): 0.70	
	Mayberry et al., 2021	Original	USA (English)	**Internal consistency:**<br>Cognitive compensation subscale (α): 0.80<br>Interpersonal Enjoyment subscale (α): 0.64<br>Frequency of Collaboration subscale (α): 0.79	No value
Scales to measure social support for diet and exercise behaviors	Sallis et al., 1986	Original and validation study	USA (English)	**Internal consistency:**<br>Each factor (α): ranged from 0.61 to 0.87<br>**Test-retest reliability:**<br>r= ranged from 0.55 to 0.86, p< 0.001	**Structural validity:**<br>EFA for each of the four scale was done: Friend Support for Eating Scale contained four factors; Family Support for Eating Scale included five factors; Friend Support for Exercise Scale included four factors and Family Support for Exercise Scale contained four factors.<br>**Concurrent validity:**<br>r= - 0.19, p< 0.01 (Positive factor- Friend Support for Eating Scale and not heart healthy/heart healthy dietary index),<br>r= - 0.27, p< 0.001 (Encouragement factor- Family Support for Eating Scale and not heart healthy/heart healthy dietary index)<br>r= 0.46, p< 0.001 (Exercising together - Friend Support for Exercise Scale and Vigorous exercise)

					r= 0.35, $p < 0.001$ (Participation and involvement - Friend Support for Exercise Scale and Vigorous exercise)<br>r= 0.23, $p < 0.001$ (Rewards and punishments - Friend Support for Exercise Scale and Vigorous exercise)<br>**Construct validity**:<br>r= 0.22, $p < 0.01$ (Involvement factor and age)<br>$p < 0.001$ (Friend and family support for exercise habits and currently exercising)<br>$p < 0.01$ (Friend support for exercise habits and trying to get more exercise)<br>$p < 0.001$ and $p < 0.05$ (Friend and family support for eating habits and trying to eat less fat)<br>$p < 0.001$ (Friend support for eating habits and trying to eat less salt)<br>$p < 0.01$ (Family support for eating habits and trying to eat less salt)<br>$p < 0.001$ and $p < 0.05$ (Friend support for eating habits and trying to eat fewer calories)<br>$p < 0.001$ and $p < 0.05$ (Family support for eating habits and trying to eat fewer calories)
	Noroozi et al., 2011	Translation	Iran (Persian)	**Internal consistency:**<br>Friend support for exercise behavior scale (α): 0.86<br>Family support for exercise behavior scale (α): 0.90	**Content validity:**<br>CVR total = 0.94<br>**Structural validity:**<br>EFA and CFA were done. Family support for exercise behavior scale resulted in a modified 3-factor correlated model resulted in better-fit indices. Friend support for exercise behavior scale. The friend support for exercise behavior scale was similar to

					the original scale and was confirmed the 1-factor model
	Heiss & Petosa, 2015	Original	USA (English)	**Internal consistency:**<br>Friend support for exercise behavior scale (α): 0.94<br>Family support for exercise behavior scale (α): 0.95	No value
	McEwen et al., 2019	Original and Translation	USA (English and Spanish)	**Internal consistency:**<br>Family Support for Healthy Eating – Encouragement subscale (α): 0.92<br>Family Support for Healthy Eating – Sabotage subscale (α): 0.91 (participants)<br>Family Support for Healthy Eating – Sabotage subscale (α): 0.70 (family members)	No value
	Al-Ghafri et al., 2021	Translation	Oman (Arabic)	**Internal consistency:**<br>Total scale (α): 0.70<br>Family support scale (α): 0.60<br>Friends support scale (α): 0.20<br>r= ranged from −0.1 to 0.4 (baseline family version inter-item correlations)<br>r= ranged from −0.2 to 0.7 (twelve months family version inter-item correlations)<br>r= ranged from −0.01 to 0.5 (baseline friends' version inter-item correlations)<br>r= ranged from −0.08 to 0.6 (twelve months friends' version inter-item correlations)<br>**Internal consistency (12 months):**<br>Family support scale (α): 0.60<br>Friends support scale (α): 0.40	**Structural validity:**<br>Factor analysis was done. Identified the presence of six factors for family version and seven (baseline) and five factors (twelve months)

Social Provision Scale (SPS)	Cutrona & Russell, 1987	Original and validation study	USA (English)	**Internal consistency:**<br>Total scale (α): 0.92<br>Subscales (α): ranged from 0.65 to 0.76	**Structural validity:**<br>CFA was done. Provided a six factors model<br><br>**Divergent validity:**<br>r= - 0.28, $p< 0.001$ (SPS and depression)<br>r= - 0.20, $p< 0.01$ (SPS and Neuroticism)<br>**Convergent validity:**<br>r= 0.35, $p< 0.001$ (SPS and Satisfaction with support)<br>r= 0.40, $p< 0.001$ (SPS and Number of supportive persons)<br>r= 0.35, $p< 0.001$ (SPS and Number of Helping Behaviors)<br>r= 0.46, $p< 0.001$ (SPS and Attitudes towards support)
	Chun et al., 2016	Translation	USA (Cantonese)	**Internal consistency:**<br>Total scale (α): 0.80	No value
	Yeung et al., 2020	Original	New Zealand (English)	**Internal consistency:**<br>Total scale (α): 0.87	No value
Social support scale for self-care in middle-aged patients (S4-MAD)	Naderimagham et al., 2012	Original and validation study	Iran (English)	**Internal consistency:**<br>Total scale (α): 0.94<br>Subscales (α): ranged from 0.88 to 0.97<br>**Test-retest:**<br>ICC: 0.87	**Content validity:**<br>CVR and CVI were calculated. A CVI score of 0.80 or above was considered satisfactory and items with CVR score of 0.56 or above were selected<br>**Structural validity:**<br>CFA and EFA were done. Was indicated a five-factor structure and was showed that factor structure of this scale was appropriate
Unsupportive social interaction scale (USIS)	Baron-epel et al., 2015	Original and validation study	Israel (Hebrew)	**Internal consistency:**<br>Total scale (α): 0.84<br>USIS interference subscale (α): 0.85<br>USIS insensitivity subscale (α): 0.73	**Structural validity:**<br>EFA was done. Was extracted two theoretical factors<br>**Convergent validity**

				r= ranged from 0.38 to 0.67 (corrected item-total)<br>r= 0.37, *p*< 0.0001 (between the two subscales)	r= 0.08, *p*< 0.013 (USIS and self-reported health)<br>r= 0.19, *p*< 0.0001 (USIS and feeling bad)<br>r= 0.16, *p*< 0.0001 (USIS and satisfaction with treatment)<br>r= 1.93, *p*< 0.006 (USIS and not smoking)<br>r= 1.83, *p*< 0.001 (USIS and physical activity)<br>**Divergent validity:**<br>r= - 0.22, *p*< 0.0001 (USIS and social support)<br>r= - 0.12, *p*< 0.0001 (USIS and frequency of meeting family)<br>r= - 0.12, *p*< 0.0001 (USIS and frequency of going out with other people)

NOTE: BDI SF: Beck Depression Inventory—Short Form; CES-D: Center for Epidemiologic Studies Depression Scale; CVI: Content Validity Index; CVR: Content Validity Ratio; DDS:Diabetes Distress Scale; EI: Emotional Involvement; FIAD: Family and Friend Involvement in Adults' Diabetes; FSS: Family Satisfaction Scale; GHb: Glycosylated hemoglobin; ICC: Intraclass correlation coefficient; IDDM: insulin dependent diabetes mellitus; IHLC: Internal Health of Control scale; IOCQ: Important Other(s) Climate Questionnaire; IOCQ-D: Important other support healthy diet; IOCQ–S: Important other support smoking cessation; IPAQ: International Physical Activity Questionnaire (short form); NIDDM: noninsulin dependent diabetes mellitus; PC: Perceived Criticism; PDSMS-4: Diabetes self-efficacy was assessed with a 4-item version of the Perceived Diabetes Self-Management Scale; PDQ: Personal Diabetes Questionnaire; PCQ: Perceptions of Collaboration Questionnaire; PDQ: Personal Diabetes Questionnaire; PDSMS: Perceived Diabetes Self-Management Scale; PRQ: Personal Resource Questionnaire; SDSCA: Summary of Diabetes Self-care Activities; SFI: Self-report Family Inventory; SF-36: Short Form 36; EFA: Exploratory factor analysis; CFA: Confirmatory factor analysis; (α): Cronbach's alpha.
